# Coherency of circadian rhythms in the SCN is governed by the interplay of two coupling factors

**DOI:** 10.1371/journal.pcbi.1006607

**Published:** 2018-12-10

**Authors:** Isao T. Tokuda, Daisuke Ono, Sato Honma, Ken-Ichi Honma, Hanspeter Herzel

**Affiliations:** 1 Department of Mechanical Engineering, Ritsumeikan University, Shiga, Japan; 2 Photonic Bioimaging Section, Research Center for Cooperative Projects, Hokkaido University Graduate School of Medicine, Sapporo, Japan; 3 Department of Chronomedicine, Hokkaido University Graduate School of Medicine, Sapporo, Japan; 4 Institute for Theoretical Biology, Charité and Humboldt University of Berlin, Berlin, Germany; Northeastern University, UNITED STATES

## Abstract

Circadian clocks are autonomous oscillators driving daily rhythms in physiology and behavior. In mammals, a network of coupled neurons in the suprachiasmatic nucleus (SCN) is entrained to environmental light-dark cycles and orchestrates the timing of peripheral organs. In each neuron, transcriptional feedbacks generate noisy oscillations. Coupling mediated by neuropeptides such as VIP and AVP lends precision and robustness to circadian rhythms. The detailed coupling mechanisms between SCN neurons are debated. We analyze organotypic SCN slices from neonatal and adult mice in wild-type and multiple knockout conditions. Different degrees of rhythmicity are quantified by pixel-level analysis of bioluminescence data. We use empirical orthogonal functions (EOFs) to characterize spatio-temporal patterns. Simulations of coupled stochastic single cell oscillators can reproduce the diversity of observed patterns. Our combination of data analysis and modeling provides deeper insight into the enormous complexity of the data: (1) Neonatal slices are typically stronger oscillators than adult slices pointing to developmental changes of coupling. (2) Wild-type slices are completely synchronized and exhibit specific spatio-temporal patterns of phases. (3) Some slices of Cry double knockouts obey impaired synchrony that can lead to co–existing rhythms (“splitting”). (4) The loss of VIP-coupling leads to desynchronized rhythms with few residual local clusters. Additional information was extracted from co–culturing slices with rhythmic neonatal wild-type SCNs. These co–culturing experiments were simulated using external forcing terms representing VIP and AVP signaling. The rescue of rhythmicity via co–culturing lead to surprising results, since a cocktail of AVP-antagonists improved synchrony. Our modeling suggests that these counter-intuitive observations are pointing to an antagonistic action of VIP and AVP coupling. Our systematic theoretical and experimental study shows that dual coupling mechanisms can explain the astonishing complexity of spatio-temporal patterns in SCN slices.

## Author summary

The mammalian circadian clock is orchestrated by a network of coupled neurons. Brain slice preparations allow the analysis of coupling mechanisms mediated by neuropeptides. From bioluminescence recordings, we extract single cell characteristics such as period, amplitude and damping rate. Our data-based stochastic network model involves local coupling between cells and additional external forcing. Available experimental data guide our simulations with two distinct coupling and forcing mechanisms representing the neuropeptides VIP and AVP. We compare our simulations with experiments from neonatal and adult wild-type brain slices and multiple knockouts. Furthermore, we study co–culturing of slices with synchronized neonatal wild-type slices. The extreme complexity of the spatio-temporal patterns is quantified using empirical orthogonal functions (EOFs). The experimental reduction of AVP coupling leads to surprising observations. In double knockouts, inhibition of AVP signaling can improve synchrony, whereas, in triple knockouts, coherency is reduced. Our network modeling shows that these counter-intuitive observations can be explained by an antagonistic action of VIP and AVP signaling. The agreement of experiments and simulations suggests that quite complex spatio-temporal patterns can appear as emergent properties of oscillator networks with dual coupling mechanisms.

## Introduction

Circadian rhythms in mammals are orchestrated by the suprachiasmatic nucleus (SCN)—a densely coupled network of about 20,000 neurons [1–3]. Gene–regulatory feedback loops generate noisy oscillations of gene expression and firing rate in individual neurons [4, 5]. Coupling of individual cells leads to synchronization [6] and to periodicity with astonishingly high precision [7]. The detailed coupling mechanisms between SCN neurons are debated. Among a variety of neuropeptides, vasoactive intestinal polypeptide (VIP) and arginine vasopressin (AVP) mediate networking in the SCN [8–11].

The SCN network generates robust self-sustained rhythms of firing rate, it can be adjusted by light inputs via the retinohypothalamic tract (RHT), and it orchestrates multiple outputs [12]. Extensive studies based on immunostaining and reporter signals revealed an enormous spatio-temporal complexity of the SCN [13, 14]. Interestingly, the network structure exhibits pronounced plasticity in development and across seasons [15–17]. Seasonal variability is associated with varying phase relationships of SCN regions modulated by the neurotransmitter GABA [18, 19].

The neuropeptide VIP is considered to be a “master synchronizer” and knockouts of VIP and its receptor lead to purely synchronized rhythms [20–22]. AVP is rhythmically regulated by the clock and it is broadly expressed in the SCN. AVP signaling can coordinate circadian cells especially in the absence of VIP [9, 23]. Loss of AVP receptors weakens the clock and accelerates re-entrainment [10].

In most studies, coupling via VIP, GABA, and AVP has been studied individually using knockouts and inhibitors. Here, we focus on the interactions of coupling agents such as VIP and AVP. It has been predicted in previous studies that in such situations phase relationships play a major role [24, 25]. It was shown experimentally that the expression levels and phases of VIP and AVP are quite variable depending on developmental stage and light conditions [26–32]. Reporter signals and expression profiles in the SCN reveal distinct rhythmicities of VIP, AVP and their receptors [33, 34]. By studying VIP and AVP mediated coupling, we address the general question on how timing of two interacting coupling mechanisms affects the synchrony and the formation of spatio-temporal patterns in the oscillator network of the SCN.

In our study, we analyze organotypic SCN slices from neonatal and adult mice in wild–type and multiple knockout conditions. Double knockouts of the core clock genes *Cry1* and *Cry2* weaken single cell rhythmicity but maintain some rhythmicity in neonatal slices [25, 35]. Knocking out in addition the VIP–receptor *Vipr2* leads to complete desynchrony. We quantify these different degrees of rhythmicity by pixel–level analysis of bioluminescence data combined with empirical orthogonal functions to extract spatio–temporal patterns [36, 37]. In order to explore the interplay of the coupling agents VIP and AVP, we study also SCN slices co–cultured with neonatal wild–type SCN slices. It has been shown earlier that such an external periodic forcing can rescue tissue–level rhythmicity [9] and that AVP signaling is critical for the restoration of circadian rhythms [11]. In order to get insight into the enormous complexity of these data, we simulate networks of oscillators with dual coupling representing the VIP and AVP. Our network model can elucidate counter-intuitive effects of the interplay of competing coupling agents.

## Materials and methods

### Ethics statement

Experiments were conducted in compliance with the rules and regulations established by the Animal Care and Use Committee of Hokkaido University.

### Animals

*Cry* double deficient (*Cry1,2*^−/−^) mice and Vip receptor 2 deficient (*Vipr2*^−/−^) mice were bred with PER2::LUC mice carrying a PER2 luciferase reporter [38]. Wild–type (*Cry1,2*^+/+^/*Vipr2*^+/+^) PER2::LUC transgenic mice on the C57BL/6J background were used as control. Mice were reared in the animal quarters in Hokkaido University, where environmental conditions were controlled (lights–on, 6:00–18:00 h; light intensity, approximately 100 lx at the bottom of cage: humidity, 60 ± 10%).

### SCN slice and dispersed cell culture

For the measurement of PER2::LUC bioluminescence from a cultured SCN slice, mice of 8–16 weeks or 2–5 days old, kept under LD condition, were euthanized between 8:00 and 16:00 by cervical dislocation and decapitated. The brain was rapidly removed and a coronal SCN slice of 150 *μ*m or 200 *μ*m was made by a microslicer (D.S.K: DTK–1000; Dosaka EM) or a tissue chopper (McIlwain). The brain slice containing the middle portion of the SCN was selected and trimmed in approximately a 2×2 mm square. The slice was cultured in air at 36.5°C with 1.2 ml Dulbecco’s modified Eagle’s medium (Invitrogen) with 0.2 mM D–luciferin K and 5% supplement solution, the composition of which was described previously [35].

For the measurement of PER2::LUC from dispersed SCN cells, the SCNs from 4–8 neonatal pups (2–5 days old wild-type and *Cry* double deficient mice) were dissected from hypothalamic slices of 400 *μ*m thick and dissociated using trypsin. Dispersed cells were plated on a 35 mm Petri dish pre–coated with 0.01% Poly–L–ornithine. The cell density was 1100±500 cells/mm^2^. The medium composition was the same as that for the slice culture, except for 5% FBS in dispersed cell culture. In the co–culture experiment, the SCN slices of 150 *μ*m thick were obtained from adult mice carrying the PER2::LUC reporter (recipient). The slice was pre-cultured for 3 or 4 days, and then co–cultured with an SCN slice from mice without the reporter system (donor). The donor SCN slice of 200 *μ*m thick was obtained from WT mice of 7 days old and pre-cultured for one day before the co–culturing. When co–cultured, the graft SCN slice was placed inside out on the surface of recipient SCN slice. Measurement of the bioluminescence was started from the beginning of culturing of the recipient SCN and continued for at least 5 days after the co–culture. AVP receptor antagonists (V1A receptor antagonist: SR49059; TOCRIS, V1B receptor antagonist: SSR149415; Axon Medchem) were dissolved in water (SR49059 and SSR149415: final 2.5 *μ*M). Water (vehicle) or antagonists were applied into the medium 5 to 7 days after co–culturing. The chemicals were either directly added to the culture medium (bath application) or dissolved in the culture medium to exchange with the whole medium in culture.

### Bioluminescence imaging

Bioluminescence at the SCN cell level in cultured slices or in dispersed cells was obtained by DM IRB (Leica), Luminoview 200 (Olympus), or Cellgraph (Atto) equipped with an EMCCD camera cooled at −80°C. The bioluminescence was measured every 60 min with an exposure time of 59 min. The pixel size was 2.3×2.3 *μ*m for DM IRB, 2.0×2.0 *μ*m for Luminoview 200, and 1.6×1.6 *μ*m for Cellgraph. For the measurement of PER2::LUC from dispersed SCN cells, bioluminescence signals were analyzed within a region of interest (ROI). The mean area of a single ROI was about 100 *μ*m^2^, comparable to the size of a single SCN cell. The bioluminescence was expressed with an average intensity of pixels involved in a ROI.

### Empirical orthogonal functions analysis

To analyze spatio–temporal dynamics of the SCN slice movie data, the method of empirical orthogonal functions (EOFs), pioneered by Edward Lorenz in the context of statistical weather prediction [36, 39], was applied. The EOFs extract coherent structures of the spatio–temporal data as empirical eigenfunctions or empirical modes [37, 40, 41]. First, we consider the bioluminescence movie data as *T* × *N* matrix

$$\begin{array}{c} A=[\begin{array}{cccc} a_{1} & a_{2} & \cdots & a_{N} \end{array}], \end{array}$$ (1)

where *N* and *T* are the number of pixels in the SCN slice image and the number of time points, respectively. Each column vector *a*_*k*_ = [*x*_*k*_(1), *x*_*k*_(2),…, *x*_*k*_(*T*)]^*T*^ represents time–sequence of the bioluminescence signal at *k*–th location of the SCN slice image. Interdependence of the dynamics at different locations can be quantified by the covariance matrix *R* = *A*^*T*^ *A*, where the (*i*, *j*)–element corresponds to covariance of the temporal patterns between locations *i* and *j*. The EOFs of the spatio–temporal data *A* are defined as the eigenvectors *e*_*i*_ of the covariance matrix *R*, sorted with respect to the size of the eigenvalues Ω_*i*_ (in descending order). Time sequence of scalar products between *t*–th bioluminescence image and *i*–th eigenvector is called the *i*–th empirical mode *c*_*i*_(*t*). For oscillator network system, spatially coherent patterns are extracted as the major empirical modes, where the normalized eigenvalues, $\left\{100\times \Omega_{i}/\sum_{j=1}^{N}\Omega_{j}\left[\%\right]:i=1,\ldots ,N\right\}$, quantify the variance of the corresponding components.

### Single cell analysis and coupled amplitude–phase oscillators

As a model for circadian cells, a generic form of self–sustained oscillators is introduced as follows [42]:

$$\frac{dx}{dt}=-\lambda \frac{x}{r}\left(r-\alpha \right)-\omega y+\xi_{x},$$ (2)

$$\frac{dy}{dt}=-\lambda \frac{y}{r}\left(r-\alpha \right)+\omega x+\xi_{y}.$$ (3)

The amplitude-phase model is described in Cartesian (*x*, *y*)–coordinates with radius $r=\sqrt{x^{2}+y^{2}}$. The system gives rise to a limit cycle attractor with amplitude *α* and frequency *ω*, where perturbed dynamics returns to the attractor with a damping ratio of λ. The limit cycle is driven by independent *Gaussian* noise *ξ*_*x*_ and *ξ*_*y*_. The single cell model has five unknown parameters {*α*, *ω*, λ, *D*_*r*_, *D*_*φ*_}, which were estimated for dispersed cell culture data by fitting the autocorrelation function of the model to that of the data [25, 42]. From the estimated parameters, the coefficient of variation CV can be computed, representing the ratio of the standard deviation of the amplitude fluctuations to the oscillator amplitude. The *CV* provides a criterion to distinguish self–sustained oscillators (*CV* < 1) from noisy damped oscillators (*CV* > 1). Detailed procedures of the parameter estimation are described in S1 Text.

By introducing local connections to the single cell models Eqs (2) and (3), which have been fitted to the dispersed data, a cellular network model of the SCN was constructed as

$$\begin{array}{ccc} \frac{dx_{i}}{dt} & = & -\lambda_{i}\frac{x_{i}}{r_{i}}\left(r_{i}-\alpha_{i}\right)-\omega_{i}y_{i}+\sum_{j\in N_{i}}K\left(x_{j}-x_{i}\right)+I_{avp}\text{sin}\left(\frac{2\pi }{24}\;t\right)\\ & & +I_{vip}\text{sin}\left(\frac{2\pi }{24}\;\left(t+\phi \right)\right)+\xi_{x,i}, \end{array}$$ (4)

$$\frac{dy_{i}}{dt}=-\lambda_{i}\frac{y_{i}}{r_{i}}\left(r_{i}-\alpha_{i}\right)+\omega_{i}x_{i}+\xi_{y,i},$$ (5)

where *x*_*i*_ and *y*_*i*_ represent dynamical variables of the *i*–th cell (*i* = 1, 2,…, *N*), $r_{i}=\sqrt{x_{i}^{2}+y_{i}^{2}}$, and *N*_*i*_ stands for neighbors of the *i*–th cell. The intercellular coupling strength was decomposed into VIP and AVP as *K* = *a*_*avp*_ *K*_*avp*_ + *a*_*vip*_ *K*_*vip*_, where *K*_*avp*_ and *K*_*vip*_ stand for default strength of the VIP and AVP couplings. For simulation of the co–culture experiment, external signals from the neonatal wild–type SCN slice (24 h oscillation period) were described by intensities *I*_*avp*_ and *I*_*vip*_ for AVP and VIP signaling, respectively, the inputs of which are phase–delayed by *ϕ*. The role of *Gaussian* noise (*ξ*_*x*,*i*_, *ξ*_*y*,*i*_) is to determine the single cell oscillation property (self-sustained or noisy damped oscillator) and to suppress the network synchrony.

To simulate various types of slices (neonate *vs*. adult, wild-type *vs*. knockout), attenuation factors, *a*_*avp*_, *a*_*vip*_, were introduced to the AVP and VIP signaling. First, it has been reported that AVP expression in the SCN was significantly reduced in the *Cry1* and *Cry2* double–knockout mice [11]. Second, VIP expression and release exhibited endogenous circadian rhythms under constant dark condition in the neonatal wild-type SCN, but not in the adult wild-type SCN [28, 30], suggesting that VIP signaling is attenuated in the natural course of development. These findings lead to the following scenario [11]: (1) Through development, the VIP coupling is attenuated in adult; (2) In *Cry1,2* double–knockout and *Cry1,2* and *Vipr2* triple–knockout mice, the AVP coupling is attenuated compared to wild–type; (3) In triple knockout, the VIP coupling is completely inactivated. The actual parameter values were selected based on the synchronization diagrams of S10 Fig, panel a–c, which show dependencies of the network synchrony on the attenuation factors.

Concerning the phase difference *ϕ*, it determines synergistic or antagonistic interaction between the VIP and AVP signaling. As explained in S1 Text, in-phase (*ϕ* = 0 h) strengthens the mutual coupling, while out-of-phase (*ϕ* = 12 h) weakens it. This can be confirmed in the synchronization diagram of S10 Fig, panel d. As a value to realize antagonistic relation between VIP and AVP, their phase difference was empirically determined as *ϕ* = 11 h. The simulation details are documented in S1 Text.

## Results

### Empirical orthogonal functions quantify rhythmicity in SCN slices

**Fig 1:**
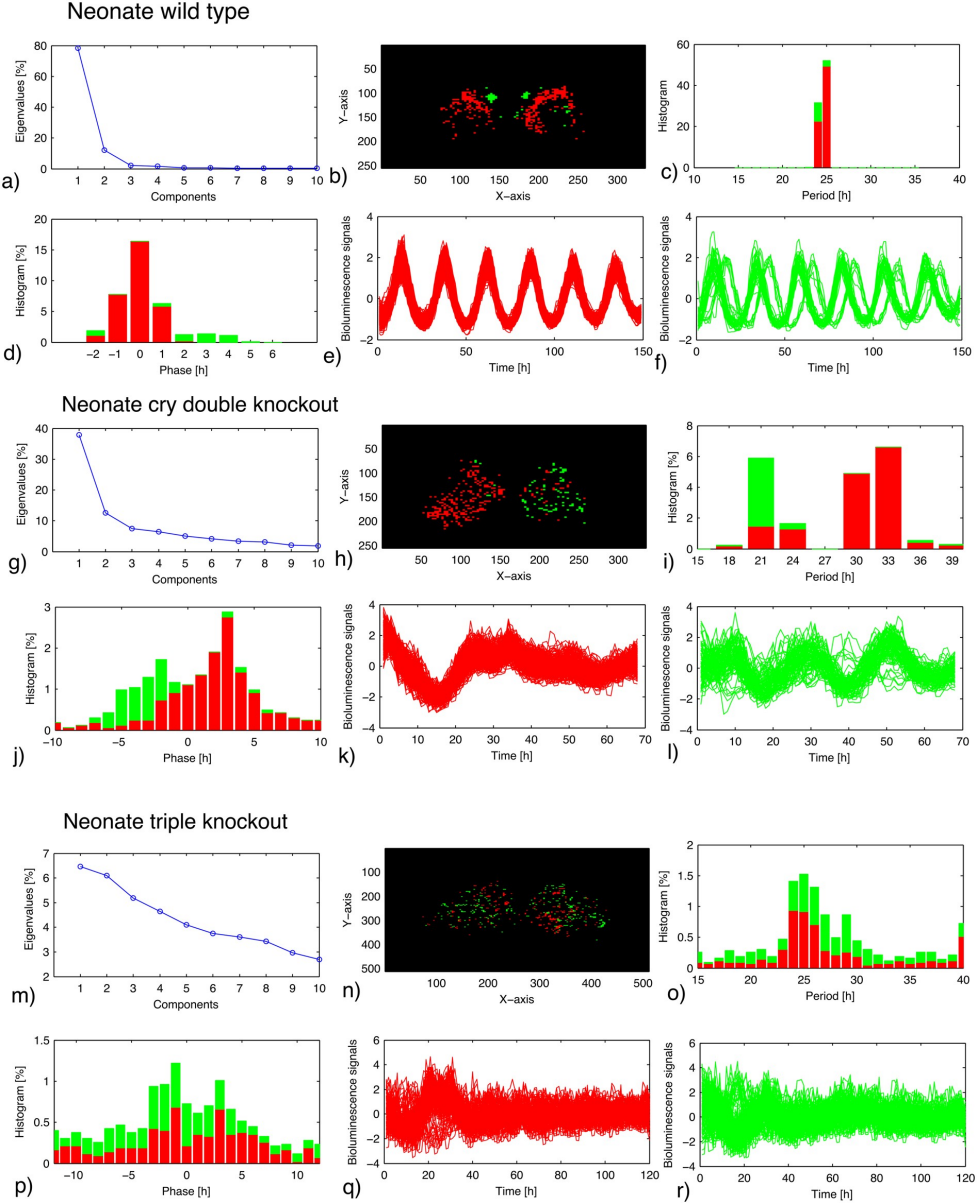
EOF analysis of PER2::LUC rhythm in the SCN of neonate wild–type mice (a–f), *Cry1* and *Cry2* double–knockout (*Cry1,2*^−/−^) mice (g–l), and *Cry1*, *Cry2*, and *Vipr2* triple–knockout (*Cry1,2*^−/−^/*Vipr2*^−/−^) mice (m–r). (**a**),(**g**),(**m**): Eigenvalues of the EOF. (**b**),(**h**),(**n**): Location of the cells classified as first (red) and second (green) components. (**c**),(**i**),(**o**): Period distribution of the cells classified as the two principal components. (**d**),(**j**),(**p**): Acrophase distribution of the cells classified as the two principal components. (**e**),(**f**),(**k**),(**l**),(**q**),(**r**): Bioluminescence traces of the cells classified as the principal components.

Synchronized rhythms of SCN neurons are particularly robust in organotypic brain slices from neonatal mice [6, 35]. In Fig 1 (upper graphs), we visualize such rhythms in a preparation from wild–type mice using PER2::LUC bioluminescence recordings. The oscillations appear totally synchronized with a period close to 24 hours and constant amplitudes over a recording time of 6 days. Such spatio–temporal patterns can be analyzed successfully by the EOFs. From the covariance matrix, the dominant spatial modes were extracted and the associated eigenvalues, representing the variance covered by these modes, were computed. Fig 1a shows that about 80% of the variance is represented by the dominant first mode (red color). Interestingly, the second mode (about 10% variance) detects also phase shifted cells in the upper part of the SCN (green). Such an advanced phase of the dorsomedial part of the SCN has been described earlier [6] and seems to be related to shorter period of cells in this area [43, 44]. Higher modes have quite small variances and provide no further information in this case. Neonatal slices typically show more phase coherent patterns than adult slices (S1 Text), pointing to developmental changes of the coupling [25, 35]. Five other slices of the neonate wild–type mice exhibited similar characteristics (sharp peak in period distribution, dominance of first and second modes, and high level of synchrony) as discussed in S1 Text and summarized in S1 Table and S1 Fig, panel d–i.

### *Cry1,2* double knockouts retain rhythmicity but may split

The locomotor activity of mice without the core clock genes *Cry1* and *Cry2* appears to be arrhythmic under constant darkness but the rhythmicity can be induced by light–dark cycles [45]. In neonatal slices of *Cry1,2* double knockouts, some remaining rhythmicities have been reported [9, 35]. However, amplitudes and periods are quite variable in different slice preparations [11]. In the middle graphs of Fig 1, we analyzed a representative example using EOFs. Here the dominant modes explain about 40% and 12% of the variance. The first mode (red) obeys a period of about 32 hours, whereas the second mode (green) oscillates with a period of about 21 hours. The spatial patterning reveals that such a splitting is induced by a desynchronization of the left and right SCNs. Note that the splitting was confirmed in two slices among eight slices of neonate *Cry1,2* double knockouts, where synchronized rhythmicities with fast damping were observed in the other six slices (S1 Text). This finding supports the hypothesis that the coupling between left and right is quite different than the coupling within the nuclei [46, 47]. Antiphase oscillations of the left and right SCNs and bimodal period distributions have been described also in hamsters and mice under constant light conditions [48, 49].

Seven slices of neonate double–knockout mice were further analyzed (see S1 Text, S1 Table, and S1 Fig, panel j–u). As described above, one slice exhibited left–right splitting (S1 Fig, panel j–o). The other six slices showed a single circadian rhythm with a global synchrony in the SCN (S1 Fig, panel p–u), being consistent with the earlier studies [35, 50]. Our interpretation is that the cellular coupling in the neonate double–knockout mice is close to the critical border. Slight difference in the coupling strength may lead either to global synchrony or to multiple clusters in the slice dynamics.

### Triple knockouts lose synchrony

As mentioned in the Introduction section, the neuropeptide VIP is a major coupling agent within the SCN. It was shown that knockouts of the neuropeptide or of its receptor *Vipr2* lead to disturbed activity rhythms and broad ranges of single cell rhythms [8]. Thus, neuronal coupling via VIP is essential to establish robust and precise rhythms. In the lower part of Fig 1, we analyzed slice data from a triple knockout, *i*.*e*., in addition to the knockouts of *Cry1* and *Cry2*, the gene for the VIP–receptor is lacking. As expected, oscillations and synchrony are largely lost. No eigenvalue exceeds 10% and individual reporter signals appear to be noisy. Still, empirical orthogonal functions can detect weak clusters with periods of about 25 hours and some spatial patterns: the red cells are primarily in the right SCN, whereas most green cells appear on the left side. Two other slices of the neonate triple knockout mice showed also a very noisy behavior (see S1 Text, S1 Table, and S2 Fig).

### Adult *Cry1,2* double knockouts lose rhythmicity

In the same manner as the neonate slices, the cultured SCN slice data from adult mice were analyzed (six slices of wild–type mice, four slices of *Cry1* and *Cry2* double–knockout mice, and four slices of *Cry1*, *Cry2*, and *Vipr2* triple–knockout mice). The results of the slice analyses are shown in detail in S1 Text and summarized in S2 Table. Representative graphs are also shown in S3 Fig.

Briefly, the adult wild–type slices showed clear circadian rhythms with global phase coherence (S3 Fig, panel a–f). Concentration of the phase–advanced pixels around innermost part of the dorsomedial SCN, observed in the neonate wild-type slice, was not recognized in the adult slice (S3 Fig, panel b), due to its slightly different configuration of the phase waves (see acrophase mapping of S1 Fig, panel c). Since phase waves and tides in the SCN are rather variable in different experimental settings, the EOF cannot be always expected to extract the same pattern of phase waves.

Adult double–knockout mice exhibited noisy and desynchronized rhythms (S3 Fig, panel g–l). As reported in [35], qualitative dynamics of the double–knockout mice changed significantly through development from neonate to adult. Adult triple–knockout mice showed even noisier behavior (S3 Fig, panel m–r). This is expected, because the VIP coupling was further diminished in the knockout slice.

To examine the four quantities (average and standard deviation of cellular periods, sum of principle eigenvalues, and synchronization index) that characterized the thirty slices from neonate and adult SCN, one-way analysis of variance (ANOVA) was carried out with respect to six groups (neonate wild-type, neonate double–knockout, neonate triple–knockout, adult wild-type, adult double–knockout, and adult triple–knockout). Statistically significant effect (*p* < 0.01) was detected for all the four quantities. According to post hoc comparisons using Fisher’s least significant difference, pairs of groups, whose means differ significantly (*p* < 0.01), were extracted. Although the results were similar among the four quantities, different pairs were also detected from one quantity to the other (see S1 Text). This indicates that the cellular periods, EOFs, and synchronization index capture similar but somewhat different features of the slice. These quantities should be utilized in a complementary fashion to detect the group differences.

### Network simulations can reproduce spatio–temporal patterns

**Fig 2:**
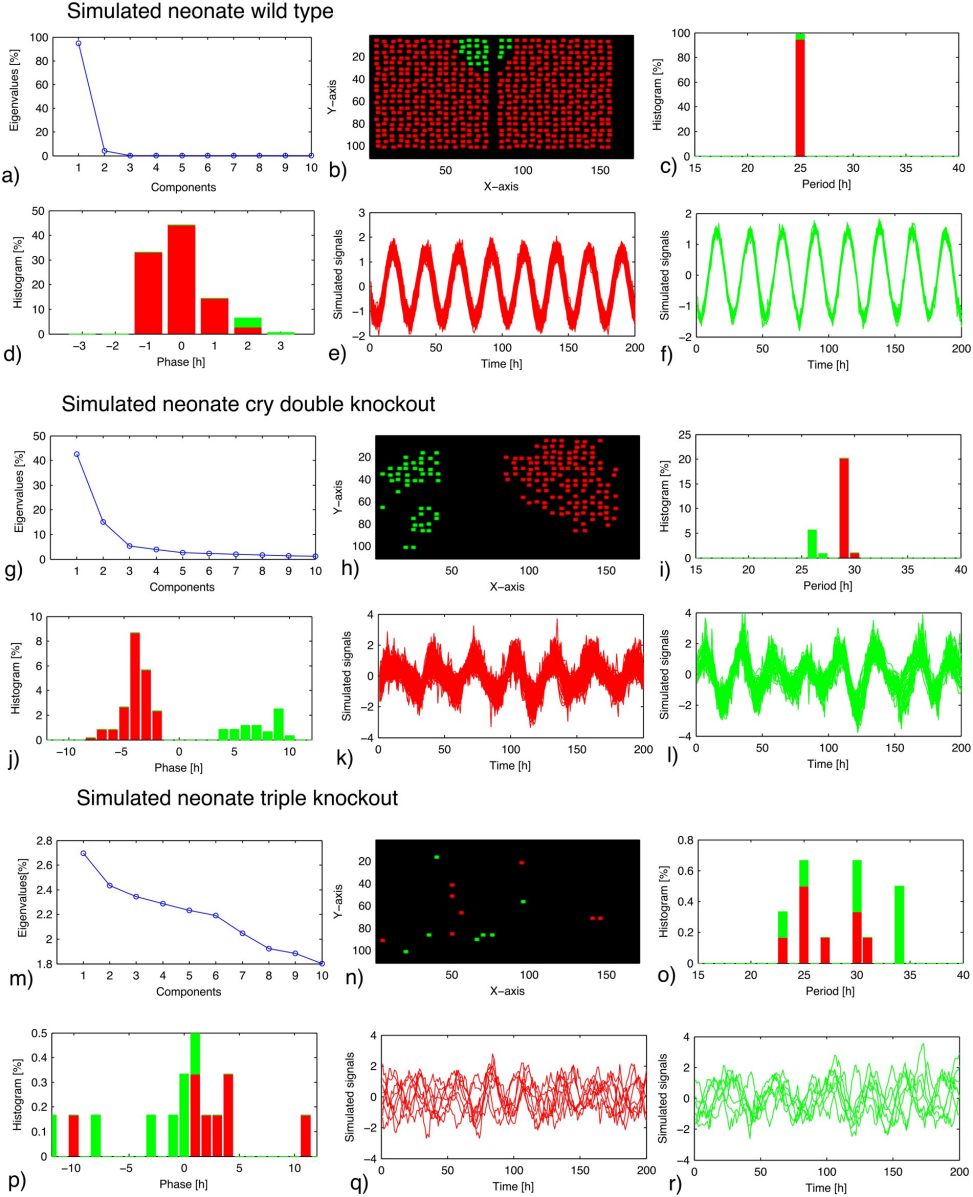
EOF analysis of simulated data for neonate wild–type mice (a–f), *Cry1* and *Cry2* double–knockout mice (g–l), and triple–knockout mice (m–r). Single cell models are based on the amplitude–phase oscillator, the parameter values of which were estimated from dispersed single cells of neonate wild–type (for wild–type simulations) and *Cry1* and *Cry2* double–knockout mice (for knockout simulations). By introducing local connections, the cellular network was simulated. In the wild–type simulation, periods of the cells located in the innermost dorsomedial SCN area are set to be lower than those of the other cells. In the knockout simulation, average periods of the cells located in the right SCN are set to be slightly larger than those of the left cells. (**a**),(**g**),(**m**): Eigenvalues of the EOF. (**b**),(**h**),(**n**): Location of the cells classified as first (red) and second (green) components. (**c**),(**i**),(**o**): Period distribution of the cells classified as the two principal components. (**d**),(**j**),(**p**): Acrophase distribution of the cells classified as the two principal components. (**e**),(**f**),(**k**),(**l**),(**q**),(**r**): Bioluminescence traces of the cells classified as the principal components.

The SCN can be regarded as a network of coupled oscillators and has been modeled extensively [24, 46, 51]. In most network models, the individual oscillator is based on transcriptional/translational feedback loops of the core clock genes [52–54]. When dealing with phenotypes displaying complex slice behaviors, such detailed biomechanical modeling approach may face difficulties, since many models might reproduce such experiments [55, 56]. Moreover, tedious optimization procedure of biochemical parameters is needed for the gene regulatory networks [56]. Our amplitude–phase model [25, 42], on the other hand, does not rely on complex gene networks. It simply connects dynamical properties of individual cells, which are quantified from dispersed cells, via inter-cellular coupling. Our former study [25] showed that such network of amplitude-phase oscillators can produce essentially the same results as those of complex gene regulatory network models. Although the amplitude–phase models do not provide a straightforward interpretation of specific gene mechanism, it has a generality of being independent of the choice of single cell models. As explained in detail in the Methods section, parameters of our single cell model in Fig 2a–2f were estimated from dispersed cells of neonate wild–type SCNs. For simulations of knockouts (Fig 2g–2r), we fitted our single cell models to SCN slices from *Cry1* and *Cry2* double–knockout mice. Our simulated cells are locally coupled via VIP and AVP terms (in Eq (4)). The corresponding coupling terms may exhibit different phases reflecting complex rhythmicities of VIP, AVP, and their corresponding receptors [8, 28, 50, 57]. Our simulations of the interplay of two different coupling terms reproduced observed counter-intuitive effects as discussed below.

Fig 2 shows that the observed spatio–temporal patterns described in Fig 1 can be simulated using the data–based stochastic single cell oscillators, local coupling, and imposed period differences. In Fig 2a–2f, we implemented the observation of Noguchi *et al*. [43] that the dorsomedial cells exhibit shorter periods. Even though all periods are locked, the second mode (green) indicates a different phase as found experimentally (compare Fig 1a–1f). Different periods of the left and right SCNs allow the simulation of splitting in Fig 2g–2l, that is comparable to the experimental data in Fig 1g–1l. Finally, we simulated triple knockouts in Fig 2m–2r by a reduced VIP–coupling and found largely random periods with small clusters that resemble the corresponding EOF analysis in Fig 1m–1r.

Our simulations in Fig 2 illustrate that rather few assumptions are required to reproduce quite complex spatio–temporal patterns in the SCN. Noisy single cell oscillators close to the Hopf bifurcation can be synchronized efficiently [44] and imposed period differences lead to phase and frequency clusters as observed experimentally.

### Periodic forcing via co–culturing can rescue synchrony

**Fig 3:**
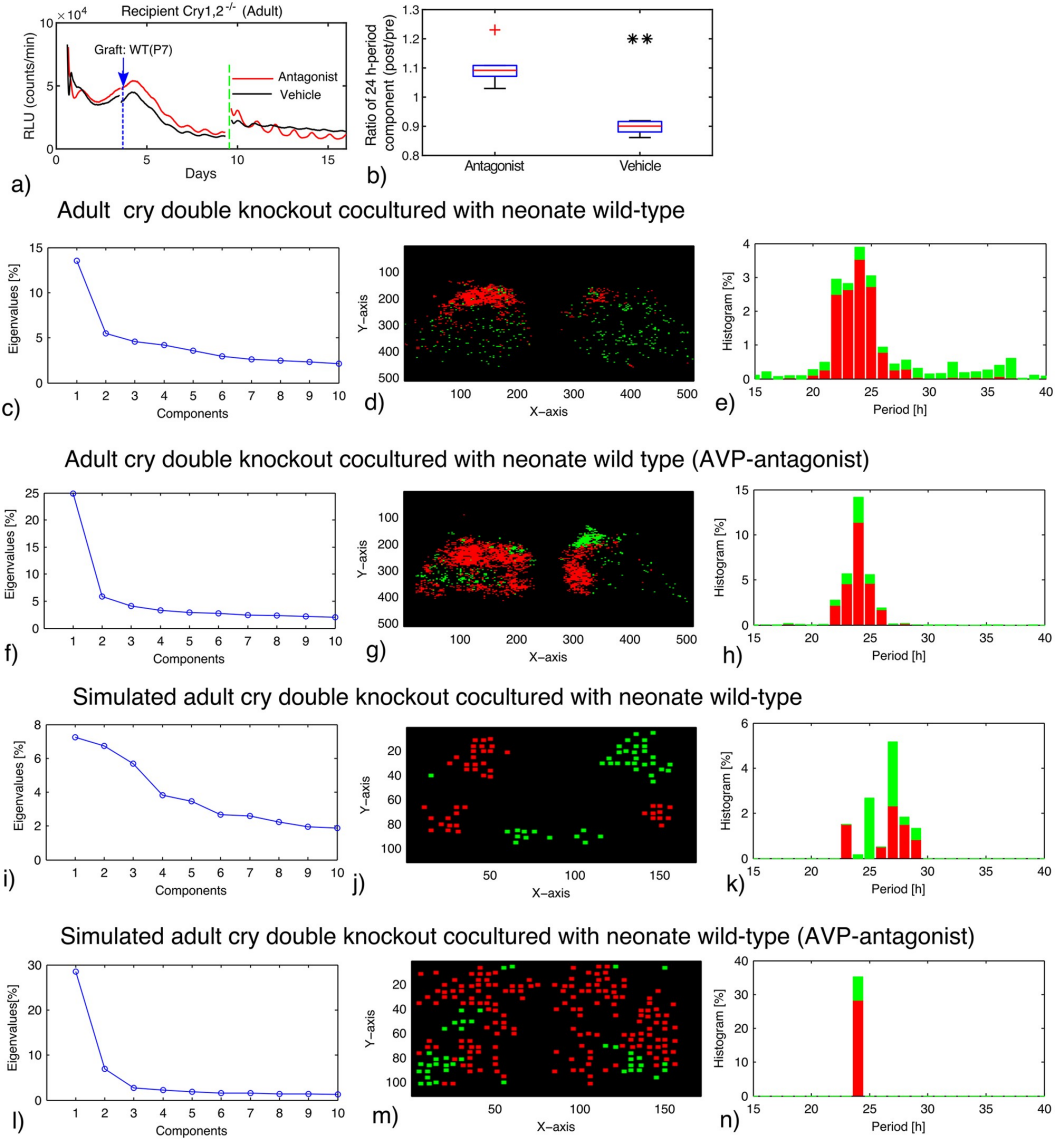
(**a**): **PER2::LUC rhythms of adult SCN slice of *Cry1* and *Cry2* double–knockout mice co–cultured with neonatal wild–type SCN slice**. After starting the co–culture (blue arrow), a cocktail of AVP receptor antagonists (SR49059: AVP receptor V1a antagonist, SSR149415: AVP receptor V1a and V1b antagonists) (red) or vehicle (black) was applied (green dotted line). (**b**): Ratio of the 24 h period component after the drug treatment to that before the treatment (*n* = 6; each for vehicle and antagonists). ** indicates significant difference (*P* < 0.01, student’s t-test) between antagonists and vehicle treatments. (**c**)-(**n**): EOF analysis of the movie data of co–cultured double–knockout slice (**c**–**h**) and the simulated data (**i**–**n**). AVP receptor antagonists were applied/simulated in in (**f**–**h**) and (**l**–**n**). Eigenvalues of the EOF (**c**,**f**,**i**,**l**), location of the cells classified as first (red) and second (green) components (**d**,**g**,**j**,**m**), and period distribution of the cells classified as the two principal components (**e**,**h**,**k**,**n**) are drawn.

Slices from adult *Cry1* and *Cry2* double knockout mice lose synchrony [25, 35]. Along the lines of Maywood *et al*. [9], SCN slices were co–cultured with neonatal wild–type SCN slices, which do not carry a bioluminescence reporter [11]. From a dynamical systems point of view, this protocol corresponds to a periodic forcing via paracrine signaling. Consequently, we extended our model by adding periodic forcing terms that represent external VIP and AVP signaling (see Eq (4)). Fig 3c–3e and S5 Fig, panel a–c,g–i, display a representative double knockout slice with co–culture. We find a partial rescue with a wide range of periods ranging from 15 to 37 hours with a broad peak around 24 hours. The dominant mode represents a variance of about 14%. In order to study the interplay of VIP and AVP coupling, a cocktail of AVP receptor antagonists has been added [11]. Unexpectedly, the cocktail enhanced significantly the amplitudes and the synchrony of adult *Cry1* and *Cry2* double knockout SCNs (see Fig 3f–3h and S5 Fig, panel d–f,j–l). The period distribution is much narrower and the dominant mode has an increased variance of 25%. The same feature was observed in two other slices (see S1 Text, S5 and S6 Figs, S3 Table). According to paired t-test applied to *n* = 3 slices, significant difference (*p* = 0.001) between control and AVP antagonists was detected using average period as the statistical quantity. Moreover, the 24 h-period component was strengthened by the antagonists treatment with a significant difference (*p* < 0.01) between antagonists and vehicle (Fig 3b). This is a counter-intuitive observation, since the weakening of coupling via AVP receptor antagonists improved synchrony.

Our modeling provides insight into the combinatorial effects of multiple coupling agents. Since the VIP and AVP coupling terms in our model exhibit different phases, their effect can by synergistic or competitive depending on their phase relationship (*ϕ* in Eq (4)). Fig 3l–3n demonstrates that the inhibition of one coupling agent can indeed improve synchrony. Thus, experimental data and simulations indicate that, in the preparations from adult double knockouts, VIP and AVP couplings act antagonistically. This explains why inhibition of AVP coupling can improve rhythmicity.

### Inhibition of VIP coupling can improve rescued rhythms

**Fig 4:**
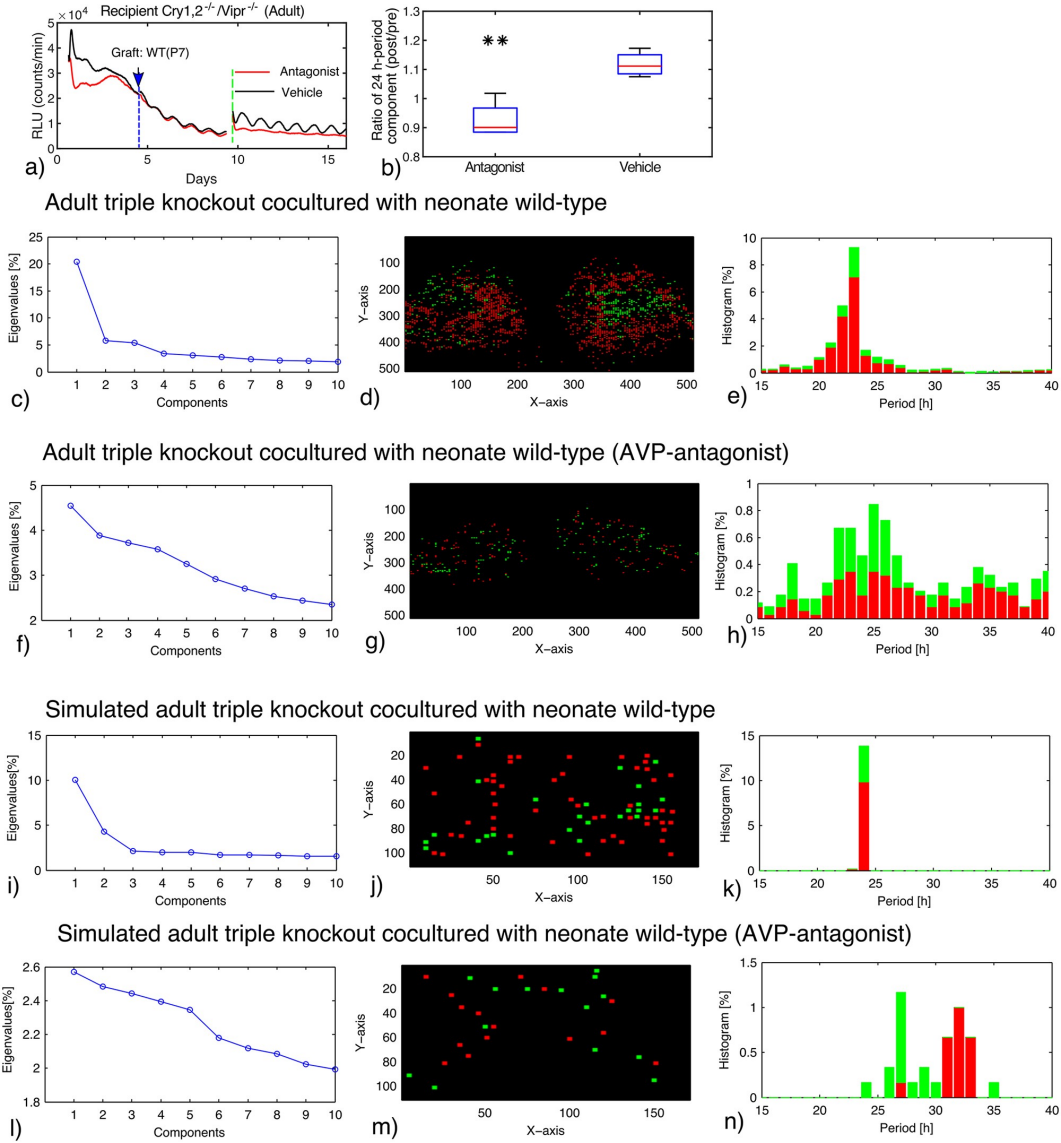
(**a**): **PER2::LUC rhythms of adult SCN slice of *Cry1*, *Cry2*, and *Vipr2* triple–knockout mice co–cultured with neonatal wild–type SCN slice. After starting the co–culture (blue arrow), a cocktail of AVP receptor antagonists (SR49059: AVP receptor V1a antagonist, SSR149415: AVP receptor V1a and V1b antagonists) (red) or vehicle (black) was applied (green dotted line)**. (**b**): Ratio of the 24 h period component after the drug treatment to that before the treatment (*n* = 4; each for vehicle and antagonists). ** indicates significant difference (*P* < 0.01, student’s t-test) between antagonists and vehicle treatments. (**c**)-(**n**): EOF analysis of the movie data of co–cultured triple–knockout slice (**c**–**h**) and the simulated data (**i**–**n**). AVP receptor antagonists were applied/simulated in in (**f**–**h**) and (**l**–**n**). Eigenvalues of the EOF (**c**,**f**,**i**,**l**), location of the cells classified as first (red) and second (green) components (**d**,**g**,**j**,**m**), and period distribution of the cells classified as the two principal components (**e**,**h**,**k**,**n**) are drawn.

If there is indeed an antagonistic relationship between VIP and AVP couplings, perturbations of VIP signaling alone might also improve rhythmicity in periodically forced SCN slices. In order to test such situations, triple knockouts of *Cry1*, *Cry2*, and the VIP receptor *Vipr2* were studied [11]. Surprisingly, the slices from triple knockout mice exhibited indeed improved rescue behavior compared to those from the double knockout mice [11]. Fig 4c–4e shows an example of such a rescued rhythmicity. The periods center around 24 hours and the first mode has a variance of more than 20%. Simulations confirmed that very weak single cell oscillators (compare Fig 1m–1r) can be synchronized efficiently with external forcing (Fig 4i–4k).

Finally, we studied the combined perturbation of both coupling agents. In Fig 4f–4h, the triple knockouts were further inhibited by the AVP antagonist. This implies that both major coupling factors were no longer acting, because the AVP signaling from the co–culture was inhibited. At the end, the synchrony was lost, a wide range of periods were observed, and all the EOFs have their variances below 5%. The reduced level of synchrony was observed also in two other slices (see supplementary S1 Text, S7 and S8 Figs, and S3 Table). The difference between control and AVP antagonists was significant (*p* = 0.02) using average period as the statistical quantity. Furthermore, the 24 h-period component was weakened by the antagonists with a significant difference (*p* < 0.01) from vehicle control (Fig 4b). Such a loss of synchrony is also visible in the associated simulations in Fig 4l–4n (also in S12 Fig, panel f,h).

## Discussions

In fluid dynamics and chaos theory, EOFs (also termed “bi-orthogonal decompositions”) have been applied successfully to quantify spatial eigenfunctions (“topos”) and temporal modes (“chronos”) [37, 58]. EOFs allow an easy visualization of spatio-temporal patterns and the eigenvalues quantify the variance of the associated modes. Alternatively, direct pixel-based quantification of periods, amplitudes, and phases has been used to characterize SCN dynamics [11, 59].

These approaches require careful noise reduction, trend-elimination, and rhythm detection. EOFs can be applied even to low quality recordings and involve implicitly separation of signals, trends, and noise. Thus, EOFs complement pixel-based techniques and provide quantification and visualization of spatio-temporal dynamics. Mathematical modeling of oscillator networks has a long tradition [60–64]. It has been shown that coupling of SCN neurons can lead to robust and synchronized rhythms [44, 51, 65]. In most models, specific coupling agents such as VIP have been studied. Inspired by our SCN slice data with VIP receptor knockouts and AVP suppression, we simulated the interplay of two coupling agents. We found that their phase relationship is a crucial parameter distinguishing between synergistic and antagonistic interactions.

Unfortunately, the phase difference of VIP and AVP signaling is difficult to specify. The available data on rhythms of VIP and AVP and their receptors are quite heterogeneous as reviewed in [22, 66]. Furthermore, the phases depend on the developmental stage, on light input, and on day-length [28, 30, 67]. Early SCN immunoassays indicate that VIP has its peak at subjective night whereas AVP is larger during the day [27, 68] consistent with recent expression profiles [34]. Moreover, the corresponding receptors Vipr2 and Avpr1a obey rhythmic expression with a peak around light onset [34]. The high variability of experimental data on peak phases suggests that also in simulations the phase difference between VIP and AVP signaling should be varied as an important model parameter.

**Fig 5:**
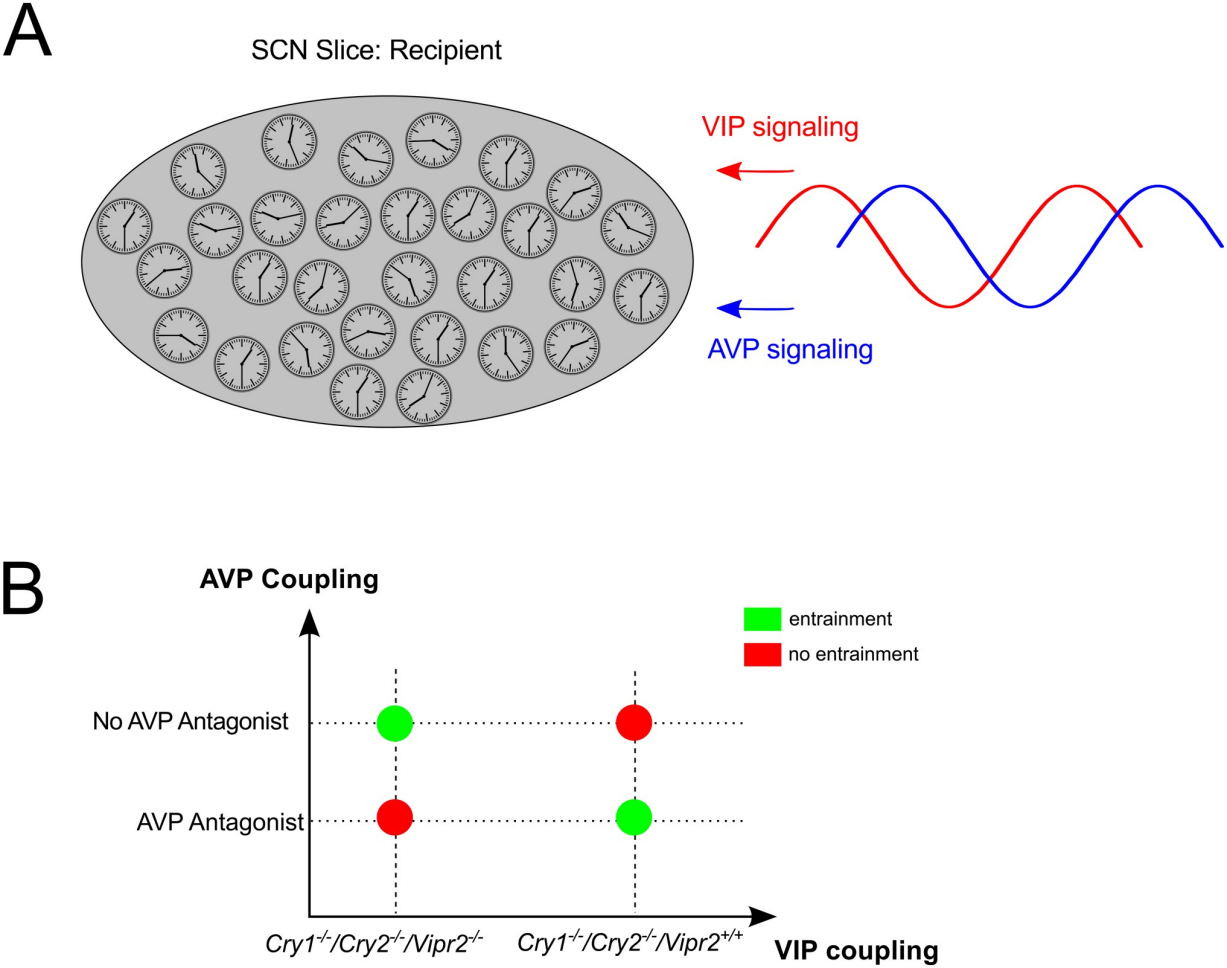
(**a**) **Schematic of the SCN network as coupled cellular oscillators. The network receives AVP and VIP signaling from graft SCN, where the signals has phase–difference (*φ*), which may produce cooperative or competing effects**. (**b**) Entrainability of the SCN slice (recipient) to neonate wild–type SCN slice (graft). The entrainment is poor when both AVP and VIP signaling coexist, implying their competing effects.

To summarize our study, EOF analysis has been applied to characterize spatio-temporal dynamics of various data including SCN slices from neonatal and adult mice, knockouts, and AVP inhibitors. EOFs extract key features of the spatio-temporal profiles of circadian gene expressions, where the variances of the dominant EOFs quantify the degree of synchronization as well as clustered dynamics. Co–culturing with wild–type neonatal slices provided further insight into the SCN slice response to external signals. Our combination of data analysis and modeling illustrates that enormous complexity of the data (see also Ono *et al*. [11, 35]) can be reproduced by simulations based on few modeling assumptions. In accordance with available data, we simulated single cells as stochastic amplitude-phase oscillators close to Hopf-bifurcations and coupled them via VIP and AVP, and periodic forcing. The diversity of mutant conditions, inhibitions and co–culturing was represented by dual coupling and forcing terms representing VIP and AVP signaling (Fig 5a). Experiments and simulations suggest that these coupling mechanisms act antagonistically (Fig 5b). From an evolutionary perspective, emergent properties due to dual coupling provide a large flexibility to the SCN network allowing fast resynchronization after jet–lag, seasonal adaptation and tuning of output signals [17, 69, 70].

## Supporting information

S1 Text Supporting information. (PDF)

S1 Fig Empirical orthogonal function analysis of PER2::LUC rhythm in the SCN of neonate wild–type mice (Slice #1: a–b, Slice #2: d–i) and *Cry1* and *Cry2* double–knockout (*Cry1,2*^−/−^) mice (Slice #2: j–o, Slice #3: p–u). (**a**): First (red) and second (green) eigenmodes of the empirical orthogonal function. (**b**),(**c**): Acrophases of whole cells on neonate wild–type slice #1 (**b**) and adult wild–type slice #1 (**c**). (**d**),(**j**),(**p**): Eigenvalues of the empirical orthogonal function. (**e**),(**k**),(**q**): Location of the cells classified as first (red) and second (green) empirical modes. (**f**),(**l**),(**r**): Period distribution of the cells classified as first (red) and second (green) empirical modes. (**g**),(**m**),(**s**): Acrophase distribution of the cells classified as first (red) and second (green) empirical modes. (**h**),(**i**),(**n**),(**o**),(**t**),(**u**): Bioluminescence traces of the cells classified as first (red) and second (green) empirical modes.
(PDF)

S2 Fig Empirical orthogonal function analysis of PER2::LUC rhythm in the SCN of neonate *Cry1, Cry2*, and *Vipr2* triple–knockout (*Cry1,2*^−/−^/*Vipr2*^−/−^) mouse (Slice #2). (**a**): Eigenvalues of the empirical orthogonal function. (**b**): Location of the cells classified as first (red) and second (green) empirical modes. (**c**): Period distribution of the cells classified as first (red) and second (green) empirical modes. (**d**): Acrophase distribution of the cells classified as first (red) and second (green) empirical modes. (**e**),(**f**): Bioluminescence traces of the cells classified as first (red) and second (green) empirical modes.
(PDF)

S3 Fig Empirical orthogonal function analysis of PER2::LUC rhythm in the SCN of adult wild–type mouse (Slice #1: a–f), adult *Cry1* and *Cry2* double–knockout mouse (Slice #1: g–l), and adult *Cry1, Cry2*, and *Vipr2* triple–knockout mouse (Slice #1: m–r). (**a**),(**g**),(**m**): Eigenvalues of the empirical orthogonal function. (**b**),(**h**),(**n**): Location of the cells classified as first (red) and second (green) empirical modes. (**c**),(**i**),(**o**): Period distribution of the cells classified as first (red) and second (green) empirical modes. (**d**),(**j**),(**p**): Acrophase distribution of the cells classified as first (red) and second (green) empirical modes. (**e**),(**f**),(**k**),(**l**),(**q**),(**r**): Bioluminescence traces of the cells classified as first (red) and second (green) empirical modes.
(PDF)

S4 Fig Average of cellular periods within slice (a), standard deviation of cellular periods within slice (b), sum of first and second eigenvalues (c), and synchronization index (d) are plotted (boxes: Average over individual slices; error-bars: Standard deviation of individual slices) for six groups (neonate wild-type: *n* = 5, neonate *Cry1* and *Cry2* double–knockout: *n* = 8, neonate *Cry1, Cry2*, and *Vipr2* triple–knockout: *n* = 3, adult wild-type: *n* = 6, adult double–knockout: *n* = 4, adult triple–knockout: *n* = 4). One-way ANOVA revealed significant main effect (*p* < 0.01) for all four quantities. Post hoc comparisons using Fisher’s least significant difference (*p* < 0.01) indicate pairs of group means that differ from each other (each pair indicated with a combination of filled circle and arrow).
(PDF)

S5 Fig Empirical orthogonal function analysis of SCN slices of adult *Cry1* and *Cry2* double–knockout mice (slice #2: a–f, slice #3: g–l) co–cultured with neonatal wild–type SCN slice. A cocktail of AVP receptor antagonists (SR49059: AVP receptor V1a antagonist, SSR149415: AVP receptor V1a and V1b antagonists) was applied to the cultured SCN slices in (**d**–**f**),(**j**–**l**). (**a**),(**d**),(**g**),(**j**): Eigenvalues of the empirical orthogonal function. (**b**),(**e**),(**h**),(**k**): Location of the cells classified as first (red) and second (green) empirical modes. (**c**),(**f**),(**i**),(**l**): Period distribution of the cells classified as first (red) and second (green) empirical modes.
(PDF)

S6 Fig Bioluminescence traces of the cells classified as first (red) and second (green) empirical modes of adult *Cry1* and *Cry2* double–knockout mice (slice #1: a,d,g,j, slice #2: b,e,h,k, slice #3: c,f,i,l) co–cultured with neonatal wild–type SCN slice. AVP receptor antagonists were applied in (**g**–**l**).
(PDF)

S7 Fig Empirical orthogonal function analysis of SCN slices of adult *Cry1, Cry2*, and *Vipr2* triple–knockout mice (slice #2: a–f, slice #3: g–l) co–cultured with neonatal wild–type SCN slice. A cocktail of AVP receptor antagonists (SR49059: AVP receptor V1a antagonist, SSR149415: AVP receptor V1a and V1b antagonists) was applied to the cultured SCN slices in (**d**–**f**),(**j**–**l**). (**a**),(**d**),(**g**),(**j**): Eigenvalues of the empirical orthogonal function. (**b**),(**e**),(**h**),(**k**): Location of the cells classified as first (red) and second (green) empirical modes. (**c**),(**f**),(**i**),(**l**): Period distribution of the cells classified as first (red) and second (green) empirical modes.
(PDF)

S8 Fig Bioluminescence traces of the cells classified as first (red) and second (green) empirical modes of adult *Cry1, Cry2*, and *Vipr2* triple–knockout mice (slice #1: a,d,g,j, slice #2: b,e,h,k, slice #3: c,f,i,l) co–cultured with neonatal wild–type SCN slice. AVP receptor antagonists were applied in (**g**–**l**).
(PDF)

S9 Fig Analysis of oscillations in dispersed SCN cell cultures for wild–type mice (a–e) and *Cry1* and *Cry2* double–knockout mice (f–j). (**a**), (**f**): Autocorrelation functions of an experimental data (red) and the corresponding amplitude–phase model (blue). (**b**), (**g**): Detrended and normalized bioluminescence signals. (**c**), (**h**): Simulated signal by the stochastic amplitude model with estimated parameters. (**d**), (**i**): Distribution of period estimated from dispersed SCN cell cultures. (**e**), (**j**): Distribution of coefficient of variation, CV, estimated from dispersed SCN cell cultures.
(PDF)

S10 Fig Synchronization analysis of the cellular network model of coupled amplitude–phase oscillators Eqs (4) and (5). (**a**): Dependence of the synchronization index *R* on the attenuation factors *a*_*vip*_∈[0, 1], *a*_*avp*_∈[0, 1] was computed for the network of wild-type cells. (**b**): Dependence of the synchronization index *R* on the attenuation factors *a*_*vip*_∈[0, 0.7], *a*_*avp*_∈[0, 1] was computed for the network of double knockout cells. (**c**): For the network of double knockout cells, synchronization indices, *R*_*l*_ and *R*_*r*_, are computed separately for left and right sides of the SCN and their average is drawn. (**d**): Entrainment property of the network of double knockout cells (*a*_*vip*_ = 0.1, *a*_*avp*_ = 0.1) forced by VIP and AVP signals *I*_*vip*_ = 0.01 and *I*_*avp*_∈[0, 0.01]. Dependence of the synchronization index *R* on the phase–delay *ϕ* and the strength of AVP signaling *I*_*avp*_ is plotted.
(PDF)

S11 Fig EOF analysis of simulated data for adult wild–type mice (a–f), *Cry1* and *Cry2* double–knockout mice (g–l), and triple–knockout mice (m–r). (**a**),(**g**),(**m**): Eigenvalues of the EOF. (**b**),(**h**),(**n**): Location of the cells classified as first (red) and second (green) components. (**c**),(**i**),(**o**): Period distribution of the cells classified as the two principal components. (**d**),(**j**),(**p**): Acrophase distribution of the cells classified as the two principal components. (**e**),(**f**),(**k**),(**l**),(**q**),(**r**): Simulated traces of the cells classified as the principal components.
(PDF)

S12 Fig Simulated traces of the cells classified as first (red) and second (green) empirical modes of for adult knockout slice co–cultured with neonatal wild–type SCN slice (double–knockout slice: (a)–(d), triple–knockout slice: (e)–(h)). Pharmacological treatment with AVP antagonists is assumed as *I*_*avp*_ = 0 in **b**),(**d**),(**f**),(**h**).
(PDF)

S1 Table Analysis of slice culture data from neonate mice (both wild–type and knockout). Average and standard deviation of the period estimated by the chi–square periodogram (significance level of 1%) [71] are indicated. Summation of the normalized first and second eigenvalues was calculated by the EOF analysis. Synchronization index *R* was also computed, where the average and standard deviation are for 24 time points.
(PDF)

S2 Table Analysis of slice culture data from adult mice (both wild–type and knockout). Average and standard deviation of the period estimated by the chi–square periodogram (significance level of 1%) [71] are indicated. Summation of the normalized first and second eigenvalues was calculated by the EOF analysis. Synchronization index *R* was also computed, where the average and standard deviation are for 24 time points.
(PDF)

S3 Table Analysis of SCN slices of adult knockout mice (*i*.*e*., recipient) co–cultured with neonate wild–type SCN slice (*i*.*e*., graft). Condition, under which a cocktail of AVP receptor antagonists was applied to the slice, was compared with the control condition. Period, estimated by the chi–square periodogram, summation of the normalized first and second eigenvalues, calculated by the EOF analysis, and synchronization index are summarized.
(PDF)
