# Supplementary material for: Coherency of circadian rhythms in the SCN is governed by the interplay of two coupling factors
Source: PLoS Comput Biol. 2018 Dec 10;14(12):e1006607. doi: 10.1371/journal.pcbi.1006607 (PMC6301697; doi:10.1371/journal.pcbi.1006607)
Supplement: S1 Text — (PDF) [file pcbi.1006607.s001.pdf]

Supporting Material for "Coherency of circadian rhythms in the SCN is governed by the interplay of two coupling factors"

Isao T. Tokuda, Daisuke Ono, Sato Honma, Ken-Ichi Honma, and Hanspeter Herzel

This supplementary material presents detailed analyses of the experimental data and numerical procedures for simulations of the cellular network model. It contains five sections: (1) Empirical orthogonal functions analysis, (2) quantification of slice data, (3) quantification of co–cultured slice data, (4) amplitude–phase modeling of dispersed data, (5) network models of coupled amplitude–phase oscillators.

1 Empirical orthogonal functions analysis

The method of empirical orthogonal functions (EOFs) [1, 2], also called the method of empirical eigenfunctions, proper orthogonal decomposition, principal component analysis, singular spectrum analysis, Karuhnen–Loéve (KL) transform or bi–orthogonal decomposition, provides an algorithm for pattern recognition by using linear correlations in space. Coherent structures, *i.e.*, empirical eigenfunctions or empirical modes, are extracted from spatio–temporal data [3, 4, 5].

First, we consider spatio–temporal movie data as $N \times T$ matrix, a column of T photos p_i or as a row of N columns a_k, each describing the dynamics at one location:

$$A = \begin{bmatrix} a_1 & a_2 & \cdots & a_N \end{bmatrix} = \begin{bmatrix} p_1 \\ p_2 \\ \vdots \\ p_T \end{bmatrix}. \tag{S1}$$

Photographic image in two–dimension (*i.e.*, bioluminescence imaging of SCN slice) can be represented as row vectors by identifying the vector components with the locations of the photos in any arbitrary order. To measure linear interdependence of the dynamics at different locations, the covariance matrix is

computed as

$$R = \begin{bmatrix} \langle a_1a_1\rangle & \langle a_1a_2\rangle & \cdots & \langle a_1a_N\rangle \\ \langle a_2a_1\rangle & \langle a_2a_2\rangle & \cdots & \langle a_2a_N\rangle \\ \vdots & \vdots & \ddots & \vdots \\ \langle a_Na_1\rangle & \langle a_Na_2\rangle & \cdots & \langle a_Na_N\rangle \end{bmatrix} = \left[\ A^T \quad A\ \right]. \tag{S2}$$

The (i, j)–element corresponds to the covariance of the temporal patterns between locations i and j, *i.e.*, $\langle a_i a_j\rangle = \frac{1}{T}\sum_{t=1}^{T} a_i(t)a_j(t)$, where the bracket $\langle\rangle$ denotes time–average and the temporal pattern at each location is assumed to have zero time–average, *i.e.*, $\langle a_i\rangle = \frac{1}{T}\sum_{t=1}^{T} a_i(t) = 0$. The EOFs of the movie A are defined as the eigenvectors e_i of the symmetric matrix R, sorted with respect to the size of the eigenvalues Ω_i (in descending order). The sequence of scalar products of the photographic images with one particular mode is called the mode amplitude $c_i(t)$ of that mode. For spatio–temporal data generated from a stochastic linear system, this method detects the normal modes of the system as empirical eigenfunctions [2, 3]. For oscillator network system, the EOFs extract spatially coherent pattern that maximizes its variance.

Figure 1a–f shows result of the EOF analysis applied to the bioluminescence movie data of a neonate wild–type SCN slice (Slice # 1). Time traces $\{a_i(t) : t = 1, \ldots, T\}$ were extracted at individual pixels. To focus only on oscillating regions in the SCN, time traces with small amplitude, *i.e.*, $\langle a_i^2\rangle^{1/2} \leq A_{th}$, were excluded from the analysis, where the threshold value A_{th} was manually determined. The EOF analysis was carried out by the `pca` subroutine of the MATLAB Statistical Toolbox. Normalized eigenvalues, $\{100\times\Omega_i/\sum_{j=1}^{N}\Omega_j[\%] : i = 1, \ldots, N\}$, shown in Figure 1a indicate that the largest eigenvalue Ω_1 dominates the oscillation modes (80%). The corresponding first empirical mode, $c_1(t)$ (red line in Figure S1a), represents a typical circadian time trace with 24.4 h period. The second empirical mode, $c_2(t)$ (green line in Figure S1a), on the other hand, shows another circadian time trace with advanced phase to the first mode.

The pixel points, the temporal patterns of which are dominated by the first or second mode, are drawn in Figure 1b (the first or second mode is considered dominant in pixels, in which the corresponding component of the eigenvector, *i.e.*, $|e_1|$ or $|e_2|$, takes the largest value). The first mode spreads over the whole SCN region (both ventrolateral and dorsomedial regions), whereas the second mode is concentrated around the innermost dorsomedial SCN. It has been known that the phase waves are initiated from the innermost dorsomedial SCN and travel to the ventrolateral SCN [6]. The acrophase mapping of slice # 1 indeed displays this feature in Figure S1b, implying that the EOF analysis captured the characteristics of phase waves. Individual time traces, which correspond to the first and the second modes, are drawn in Figure 1d and Figure 1e, respectively. The traces of the second mode are indeed phase–advanced to those of the first mode. This can be also confirmed from the phase distribution shown in Figure 1c (red: first mode, green: second mode).

2 Quantification of slice data

2.1 Neonate slice data

The cultured SCN slice data from neonate mice were analyzed (five slices of wild–type mice, eight slices of *Cry1* and *Cry2* double–knockout mice, and three slices of *Cry1*, *Cry2*, and *Vipr2* triple–knockout mice). From each slice, time traces $\{a_i(t) : t = 1, \ldots, T; i = 1, \ldots, N\}$ with large amplitude (*.i.e.*, $\langle a_i^2 \rangle^{1/2} > A_{th}$) were extracted on pixel basis. The extracted signals were then analyzed by the chi–square periodogram (significance level of 1 %) [7]. To quantify the level of synchrony among cellular signals within the slice, synchronization index R [8] was computed as

$$R = \frac{\langle \bar{a}^2 \rangle - \langle \bar{a} \rangle^2}{\frac{1}{N}\sum_{i=1}^{N}(\langle a_i^2 \rangle - \langle a_i \rangle^2)}, \quad \text{(S3)}$$

where the bracket $\langle\rangle$ denotes time–average and $\bar{a}$ stands for averaged variable over the cells, *i.e.*, $\bar{a}(t) = \frac{1}{N}\sum_{i=1}^{N} a_i(t)$. In the same manner as described in the previous section, the EOF analysis was further carried out. The results of the slice analyses (period estimates, synchronization index, and summation of normalized first and second eigenvalues) are summarized in Table S1.

Slice # 2 of the wild–type mouse (Figure S1d–i) showed qualitatively similar dynamics described for slice # 1 in the main text (Figure 1af). Both first and second modes, which dominate the slice dynamics ($\{\Omega_1 + \Omega_2\} > 80$ %), showed a clear circadian peak in the histogram. The pixel–based traces were well synchronized with each other ($R = 0.86 \pm 0.02$), whereas the phase was slightly advanced in the second modes located around innermost part of the dorsomedial SCN. Other four slices of the wild–type mouse exhibited similar characteristics (sharp peak in period distribution, dominance of first and second modes, and high level of synchrony) as can be confirmed in Table S1.

As shown in Figure S1j–o, slice # 2 of the *Cry1* and *Cry2* double–knockout mouse exhibited two–clusters, which were separated between left and right sides of the SCN (Figure S1k). Compared to the slice # 1 described in the main text (Figure 1i), periods did not show a clear bimodal distribution in Figure S1l. However, the phases were clearly separated between the first and second empirical modes (Figure S1m). Among the eight slices of *Cry1* and *Cry2* double–knockout mice, slices #1 and #2 showed this kind of clustering dynamics. Other six slices of the neonate double–knockout mice, on the other hand, showed a single cluster with a sharp circadian peak in the period distribution. The representative result of the EOF analysis is displayed in Figure S1p–u. These six slices were consistent with the earlier report [9, 10] that neonate *Cry1* and *Cry2* double–knockout mouse maintains a clear circadian rhythms with a global synchrony in the SCN. Our interpretation is that the cellular coupling in the neonate double–knockout mice was close to the critical value (near desynchrony). Slight difference in the coupling strength may lead either to global synchrony or to multiple clusters of the slice dynamics.

Finally, slice # 2 of the neonate *Cry1*, *Cry2*, and *Vipr2* triple–knockout mouse (Figure S2) showed a noisy behavior. The first and second empirical modes explained only 10.4 % of the slice dynamics. The periods were broadly distributed (27.7±8.1 h) and the cellular rhythms were asynchronous ($R = 0.12 \pm 0.03$). The overall characteristics were the same as those of triple–knockout slice # 1 described in the main text (Figure 1m–r). As confirmed in Table S1, the slice # 3 also had a very noisy characteristic.

2.2 Adult slice data

I the same manner as in the previous subsection, the cultured SCN slice data from adult mice were analyzed (six slices of wild–type mice, four slices of *Cry1* and *Cry2* double–knockout mice, and four slices of *Cry1*, *Cry2*, and *Vipr2* triple–knockout mice). The results of the slice analyses (period estimates, synchronization index, and summation of normalized first and second eigenvalues) are summarized in Table S2. Representative graphs are also shown in Figure S3.

Slice # 1 of the adult wild–type mouse (Figure S3a–f) showed clear circadian rhythms. The first and second empirical modes dominated the slice dynamics, while the period distribution sharply concentrated at 23.9 h. The pixel–based traces were well synchronized with each other ($R = 0.81 \pm 0.03$). The other five slices of the adult wild–type mice exhibited similar characteristics as confirmed in Table S2.

It should be noted, in Figure S3b, that the second mode with phase–advanced pixels is scattered out side of the adult slice # 1. This is in contrast to the neonate wild-type slices # 1 & # 2, in which the second mode with phase–advanced regions concentrated around innermost part of the dorsomedial SCN. The scattered location of the second mode is, however, due to the inherent property of the adult wild-type slice # 1. As displayed in the acrophase mapping of Figure S1c, the phase advanced pixels spread out side of the SCN and the EOFs extracted such structure in the movie data. Since phase waves and tides in the SCN are rather variable in different experimental settings, the EOF can not be always expected to extract the same pattern of phase waves.

Slice # 1 of the adult *Cry1* and *Cry2* double–knockout mouse (Figure S3g–l) exhibited noisy rhythms. The first and second empirical modes explained merely less than half of the slice dynamics, while the periods were broadly distributed (30.0±8.2 h) with their average deviated from 24 h. The level of synchrony was low ($R = 0.25 \pm 0.08$). Other three slices of the adult double–knockout mice also showed noisy and desynchronized rhythms. As reported in [10], qualitative dynamics of the double–knockout mice changed significantly through development from neonate to adult.

Slice # 1 of the adult *Cry1*, *Cry2*, and *Vipr2* triple–knockout mouse (Figure S3m–r) showed another noisy behavior. The first and second empirical modes explained only 28.3 % of the slice dynamics. The periods were broadly distributed (24.9±7.4 h) and the cellular rhythms were asynchronous ($R = 0.29 \pm 0.07$). As confirmed in Table S2, other three slices of the adult triple–knockout mice showed also very noisy characteristics. For the triple–knockout

mice, the qualitative dynamics remained the same through development from neonate to adult.

2.3 Statistical test

One-way analysis of variance (ANOVA) was used to examine how the slice properties, analyzed in the previous subsections, depend upon the development and phenotype. As the statistical quantity, mean period, standard deviation of cellular periods, sum of principle eigenvalues ($\Omega_1 + \Omega_2$), and synchronization index, summarized in Tables. S1 & S2, were examined. The one-way ANOVA revealed statistically significant main effect ($p < 0.01$) for all four quantities. Then, post hoc comparisons using Fisher's least significant difference ($p < 0.01$) determined which pairs of the six group means differ from each other. As shown in Figure S4, same pairs were detected as different groups for the standard deviation of cellular periods and the synchronization index, which quantify similar properties of the slice. While slightly different but similar groups were extracted for the sum of principle eigenvalues, few pairs were identified as different groups for the mean period. This indicates that the four quantities capture similar but somewhat different features of the SCN slice. Such quantities should be utilized in a complementary fashion to detect the group differences.

3 Analysis of co–cultured slice data

SCN slices of adult knockout mice (three slices of *Cry1* and *Cry2* double–knockout mice and three slices of *Cry1*, *Cry2*, and *Vipr2* triple–knockout mice) co–cultured with neonatal wild–type SCN slice were analyzed and summarized in Table S3. Pharmacological condition, under which a cocktail of AVP receptor antagonists (SR49059: AVP receptor V1a antagonist, SSR149415: AVP receptor V1a and V1b antagonists) was applied to the cultured SCN slices, was compared to the control condition. In the same manner as in the previous section, pixel–based time trace data were analyzed by the chi–square periodogram. Spatio–temporal dynamics of the slices were characterized by the EOF analysis, while the synchronization index R was computed among the cellular signals.

As shown in Figure S5a–c,g–i, slices # 2 and # 3 of the *Cry1* and *Cry2* double–knockout exhibited a broad distribution of periods without AVP receptor antagonists. With the AVP antagonists (Figure S5d–f,j–l), on the other hand, the period distribution forms a sharp circadian peak with an enhanced synchrony. This feature is consistent with the slice # 1 of the double–knockout mouse described in the main text (Figure 3c–h). Figure S6 shows bioluminescence traces of the cells classified as the principal components in the three slices. Noisy feature of the traces discernible in the first principal component of slices # 1 and # 3 (Figure S6a,c) is reduced with the AVP antagonists (Figure S6g,i). In the second principal component of slice # 2, circadian structure appears after the AVP antagonists were applied (Figure S6k).

Figure S7a–c,g–i shows that slices # 2 and # 3 of the *Cry1*, *Cry2*, and

Vipr2 triple–knockout mice gave rise to relatively a sharp circadian peak in the period distribution without AVP receptor antagonists. The level of synchrony was relatively high. With the AVP antagonists, the period distribution became broad and the synchrony was destroyed (Figure S7d–f,j–l). This is consistent with slice # 1 of the triple–knockout mouse described in the main text (Figure 4c–h). Figure S8 shows bioluminescence traces of the cells classified as the principal components in the three slices. Circadian rhythmicity recognized in the traces of slices # 1 and # 2 is lost by the administration of AVP antagonists, giving rise to noisy traces in Figure S8g–h,j–k. Although the effect of AVP antagonists is not visually recognizable in the bioluminescence traces of slice # 3 (Figure S8i,l), the effect can be quantitatively confirmed as indicated in Table S3.

According to paired t-test applied to $n = 3$ slices, significant difference between control and AVP antagonists was detected for the mean period ($p = 0.001$ for double knockout; $p = 0.02$ for triple knockout). For other quantities, the difference was rather small (standard deviation of cellular periods: $p = 0.08, 0.04$; sum of principle eigenvalues: $p = 0.25, 0.17$, synchronization index: $p = 0.04, 0.08$), because of the relatively large slice-to-slice variability.

4 Amplitude–phase modeling of dispersed data

This section provides single cell analysis of the dispersed SCN cell culture data. As a generic model for single cell oscillator, we introduce the following stochastic differential equations [11]:

$$\frac{dr}{dt} = \lambda(\alpha - r) + \xi_r, \tag{S4}$$

$$\frac{d\varphi}{dt} = \omega + \xi_\varphi. \tag{S5}$$

The system is described in polar coordinates of radius r and angle φ and has a limit cycle with amplitude α and angular frequency ω. Any perturbation away from the limit cycle will relax back with a damping rate λ. The model has two noise sources, *i.e.*, amplitude noise ($\langle \xi_r \rangle = 0$, $\langle \xi_r(t+\tau)\xi_r(t)\rangle = 2D_r\delta(\tau)$) and phase noise ($\langle \xi_\varphi \rangle = 0$, $\langle \xi_\varphi(t+\tau)\xi_\varphi(t)\rangle = 2D_\varphi\delta(\tau)$). The single cell model can be represented in Cartesian (x, y)–coordinates as

$$\frac{dx}{dt} = -\lambda\frac{x}{r}(r - \alpha) - \omega y + \xi_x, \tag{S6}$$

$$\frac{dy}{dt} = -\lambda\frac{y}{r}(r - \alpha) + \omega x + \xi_y, \tag{S7}$$

where ξ_x and ξ_y are independent *Gaussian* noises and $r = \sqrt{x^2 + y^2}$. The amplitude–phase model provides one of the simplest mathematical systems to generate limit cycle oscillations. Winfree [12] has studied this model in the context of circadian rhythms. It has been shown analytically that the stochastic

system has the following autocorrelation function [11]:

$$C(\tau) = \frac{1}{2}(\alpha^2 + \frac{D_r}{\lambda}e^{-\lambda\tau})e^{-D_\varphi\tau}\cos\omega\tau. \tag{S8}$$

The stochastic amplitude–phase model (S4),(S5) has five unknown parameters $\{\alpha, \omega, \lambda, D_r, D_\varphi\}$. Experimental data measured from dispersed SCN cell culture were fitted to the single cell model by optimizing the five unknown parameters as follows. First, bioluminescence signal recorded from dispersed SCN cell was detrended using a least square fitting of a second–degree polynomial. The polynomial was subtracted from the signal so that the signal was mean centered. The detrended signal was then normalized in such a way that the signal has zero mean and unit variance. Figure S9b,g represents detrended and normalized signals $\{z_t : t = 1, 2, \ldots, T\}$ from wild–type and *Cry1* and *Cry2* double–knockout mice, respectively.

Second, with respect to the normalized signal $\{z_t\}$, the autocorrelation $C(k)$ (with the time lag of k sampling intervals) was computed as

$$C(k) = \frac{1}{M-k}\sum_{t=1}^{M-k}(z_t - \bar{z}_0)(z_{t+k} - \bar{z}_k), \tag{S9}$$

where M is the number of samples in the time–series, and $\bar{z}_0 = \frac{1}{M}\sum_{t=1}^{M} z_t$ and $\bar{z}_k = \frac{1}{M-k}\sum_{t=1}^{M-k} z_{t+k}$ represent the mean values.

Third, the five parameters $\{\alpha, \omega, \lambda, D_r, D_\varphi\}$ of the stochastic amplitude–phase model were optimized so that the autocorrelation function (S8) of the single cell model is fitted to that of the bioluminescence signal (S9). We used `lsqcurvefit` subroutine of the MATLAB Statistical Toolbox to optimize the parameters. It should be noted that the fitting of Eq. (S8) may face the problem of local minima, since the function involves a summation of two exponentials, which may create redundancy. To avoid miss–fitting, initial guesses were used as described in detail in [11]. Figure S9a,f compares autocorrelation function $C(k)$ of the experimental data (red) and that of the fitted model (blue). The model captured basic feature of the experimental curve fairly well. Simulated traces of the stochastic amplitude–phase model (Figure S9c,h) resembled the original traces (Figure S9b,g).

From the estimated parameters, the intrinsic period and the coefficient of variation could be obtained as $T = 2\pi/\omega$ and $\mathrm{CV} = \sigma_r/\alpha = (\sqrt{D_r/\lambda})/\alpha$, where CV represents ratio of the standard deviation of the amplitude fluctuations to the amplitude of the single cell oscillator. For the experimental data shown in Figure S9b,g, they were estimated as $T = 24.5$ h, CV = 0.87 for the wild–type cell and $T = 39.5$ h, CV = 7.7 for the knockout cell. The same procedure was repeated for dispersed culture data of wild–type cells ($N = 74$) and double-knockout cells ($N = 48$). $N = 66$ WT cells and $N = 14$ knockout cells were fitted to the model reasonably well. The results are summarized in Figure S9d–e,i–j. Compared to the wild–type, the period was distributed more broadly in the knockout. The mean and the standard deviation were 24.3±1.1 h for the wild–type and 31.1±7.3 h for the double knockout; they were significantly different (t-test $p < 0.01$). Of special note is that, if the CV is greater than 1,

probability density of the state variables of the oscillator becomes qualitatively the same as that of a damped oscillator. However, if the CV is less than 1, it can be characterized as a self–sustained oscillator. Hence, $CV = 1$ provides a criterion to distinguish self–sustained oscillators from damped oscillators. Among the wild–type cells, 36.4% of the neurons were characterized as self-sustained oscillators (*i.e.*, CV < 1). Such a mixture of rhythmic and noisy cells has been described for dispersed neurons in multiple studies [11, 13, 14]. Among the knockout cells, on the other hand, no cells were classified into self–sustained oscillators.

5 Network of coupled amplitude–phase models

Following the procedure described in [14], a cellular network model of the SCN was constructed by coupling the amplitude–phase oscillators introduced in the previous section. The single cell models (S6),(S7) were locally coupled as

$$\begin{aligned}\frac{dx_i}{dt} &= -\lambda_i \frac{x_i}{r_i}(r_i - \alpha_i) - \omega_i y_i + \sum_{j \in N_i} K(x_j - x_i) + I_{avp}\sin(\frac{2\pi}{24}\ t) \\ &\quad + I_{vip}\sin(\frac{2\pi}{24}\ (t + \phi)) + \xi_{x,i}, \qquad \text{(S10)} \\ \frac{dy_i}{dt} &= -\lambda_i \frac{y_i}{r_i}(r_i - \alpha_i) + \omega_i x_i + \xi_{y,i}, \qquad \text{(S11)}\end{aligned}$$

where x_i and y_i represent dynamical variables of the i–th cell ($i = 1, 2, \ldots, N$), $\xi_{x,i}$ and $\xi_{y,i}$ are independent *Gaussian* noises, and $r_i = \sqrt{x_i^2 + y_i^2}$. N_i stands for neighbors of the i–th cell. The intercellular coupling strength was decomposed into VIP and AVP as $K = a_{avp}\ K_{avp} + a_{vip}\ K_{vip}$, where a_{avp} and a_{vip} represent attenuation factors that take into account the effect of developmental change, knockout, and pharmacological treatment. For simulation of the co–culture experiment, external signals from the neonatal wild–type SCN slice (24 h oscillation period) were described with intensities I_{avp} and I_{vip} for AVP and VIP, respectively, the inputs of which are phase–delayed by ϕ.

Values of the single cell parameters, α_i, ω_i, and λ_i, were chosen randomly from the range of estimated values for the wild-type and double knockout in the previous section. The level of synchrony was measured by the synchronization index $R = \frac{\langle \bar{x}^2 \rangle - \langle \bar{x} \rangle^2}{\frac{1}{N}\sum_{i=1}^{N}(\langle x_i^2 \rangle - \langle x_i \rangle^2)}$ as defined by Eq. (S3). $\langle \cdots \rangle$ denotes average over time and $\bar{X} = \frac{1}{N}\sum_{i=1}^{N} X_i$ is the average of x-variables among N oscillators.

To simulate the SCN cellular network, two–dimensional square lattice composed of 15×20 points was created for both left and right sides of the SCN. The cells are located on the lattice points that are apart from each other with a distance of 500±100 μm in both directions. The left and right sides of the SCN were located 1000 μm apart from each other. The cells within the distance of 1000 μm are coupled to each other as the neighbors (N_i). The coupling constants were set to $K_{vip} = 0.72$ and $K_{avp} = 0.24$ for VIP and AVP signals, respectively. Figure S10a–c shows dependence of the network synchrony

on the attenuation factors, a_{vip} and a_{avp}. For simplicity, no noise was injected. From panel (a) to (c), no external signals were applied (*i.e.*, $I_{avp} = I_{vip} = 0$). In panel (a), the network of wild-type cells was simulated for $a_{vip}{\in}[0, 1]$, $a_{avp}{\in}[0, 1]$. Strong global synchrony of $R{\approx}1$ is observed for large attenuation factors. As the attenuation factors are decreased, the level of synchrony decreases, where desynchronized cellular rhythms appear. Panels (b) shows the network of double knockout cells in $a_{vip}{\in}[0, 1.2]$, $a_{avp}{\in}[0, 1.2]$. Although strong global synchrony is observed when both attenuation factors are large, desynchronized rhythms with low synchronization index is observed in a wide range of small attenuation factors. In panels (c), on the other hand, synchronization indices, R_l and R_r, were computed separately for left and right sides of the SCN and their average was drawn for the network of double knockout cells. The wide range of desynchrony discernible in panel (b) disappears, implying that the cell are synchronized within each side of the SCN and two clusters of synchronized cells are formed. The two clusters are unified into a single cluster as the two attenuation factors become large enough.

In panel (d), external signals were applied to the network of double knockout cells coupled with attenuation factors $a_{vip} = 0.1$, $a_{avp} = 0.1$. The synchronization index R was computed by varying the strength of AVP signaling $I_{avp}{\in}[0, 0.01]$ and the phase–delay $\phi{\in}[0h, 12h]$, whereas the VIP signaling was set to $I_{vip} = 0.01$. For small phase–delay ϕ, the cells get entrained to the external signal as the AVP signaling I_{avp} is increased. For large phase–delay close to $\phi{\approx}12$ h, opposite effect of the AVP was observed. Namely, as the AVP signaling I_{avp} is increased, the level of synchrony was decreased. In Eq. (S10), the forcing term can be expressed as $I_{avp}\sin(\frac{2\pi}{24}\ t) + I_{vip}\sin(\frac{2\pi}{24}\ (t + \phi)) = I_*\sin(\frac{2\pi}{24}\ t + \phi_*)$, where $I_* = [I_{avp}^2 + I_{vip}^2 + 2I_{avp}I_{vip}\cos\frac{2\pi\phi}{24}]^{1/2}$ and $\phi_* = \tan^{-1}(\frac{I_{vip}\sin(2\pi\phi/24)}{I_{avp}+I_{vip}\cos(2\pi\phi/24)})$. The forcing strength I_* is enhanced, *i.e.*, $I_* = I_{avp} + I_{vip}$, when VIP and AVP signals are in–phase ($\phi = 0$), whereas it is suppressed, *i.e.*, $I_* = |I_{avp} - I_{vip}|$, when VIP and AVP signals are out of phase ($\phi = 12$h). By this way, the competing effect between the AVP and VIP signaling can be modeled.

To simulate various types of slices (neonate *v.s* adult, wild-type *v.s* knockout), attenuation factors (a_{avp}, a_{vip}) for the AVP and VIP signaling were determined. First, it has been reported that AVP expression in the SCN was significantly reduced in the *Cry1* and *Cry2* double–knockout mice [15]. Second, VIP expression and release exhibited endogenous circadian rhythms under constant dark condition in the neonatal wild-type SCN, but not in the adult wild-type SCN [16, 17], suggesting that VIP signaling is attenuated in the natural course of development. These findings lead to the following scenario [15]. (1) Through development, the VIP coupling is attenuated as $a_{vip} = 0.1$ in adult, while $a_{vip} = 1$ for neonate. (2) In *Cry1* and *Cry2* double–knockout mice (and triple knockout mice), the AVP coupling is attenuated as $a_{avp} = 0.1$, while $a_{avp} = 1$ for wild–type. (3) For triple knockout, the VIP coupling is completely inactivated as $a_{vip} = 0$.

Based upon these conditions, three types of slices were simulated for neonate

wild–type (Figure 2a–f), neonate *Cry1* and *Cry2* double–knockout (Figure 2g–l), and neonate *Cry1*, *Cry2*, and *Vipr2* triple–knockout mice (Figure 2m–r). To imitate the phase–wave, which are initiated from the of the dorsomedial SCN, intrinsic periods ($2\pi/\omega_i$) of the cells located in the innermost dorsomedial region were shortened by 20 %. In addition to the neonate slice, three types of slices were simulated for adult mice (wild–type: Figure S11a–f; *Cry1* and *Cry2* double–knockout: Figure S11g–l; *Cry1*, *Cry2*, and *Vipr2* triple–knockout: Figure S11m–r). Locations of the corresponding parameter settings are indicated in the synchronization diagram of Figure S10a–c (circles: neonate, squares: adult).

The co–culture experiments were further simulated for adult *Cry1* and *Cry2* double–knockout mice (Figure 3i–n) and adult *Cry1*, *Cry2*, and *Vipr2* triple–knockout mice (Figure 4i–n). In these simulations, strength of the external signals were set as $I_{avp} = 0.01$ and $I_{vip} = 0.01$ for AVP and VIP, respectively, with their phase difference of $\phi = 10$ h. Locations of the corresponding parameter settings are indicated in the synchronization diagram of Figure S10d. For the triple–knockout mice, the VIP signaling was inactivated ($I_{vip} = 0$). In experiment, under which AVP receptor antagonists were applied, the AVP signaling was blocked ($I_{avp} = 0$).

References

[1] Edward N Lorenz. *Empirical orthogonal functions and statistical weather prediction*. Massachusetts Institute of Technology, Department of Meteorology, 1956.

[2] A Hannachi, IT Jolliffe, and DB Stephenson. Empirical orthogonal functions and related techniques in atmospheric science: A review. *International journal of climatology*, 27(9):1119–1152, 2007.

[3] Gerald R North. Empirical orthogonal functions and normal modes. *Journal of the Atmospheric Sciences*, 41(5):879–887, 1984.

[4] David A Berry, Hanspeter Herzel, Ingo R Titze, and Katharina Krischer. Interpretation of biomechanical simulations of normal and chaotic vocal fold oscillations with empirical eigenfunctions. *The Journal of the Acoustical Society of America*, 95(6):3595–3604, 1994.

[5] Jörg Arndt, Hanspeter Herzel, Sumit Bose, Martin Falcke, and Eckehard Schöll. Quantification of transients using empirical orthogonal functions. *Chaos, Solitons & Fractals*, 8(12):1911–1920, 1997.

[6] Hirokazu Fukuda, Isao Tokuda, Seiichi Hashimoto, and Naoto Hayasaka. Quantitative analysis of phase wave of gene expression in the mammalian central circadian clock network. *PLoS One*, 6(8):e23568, 2011.

[7] Phillip G. Sokolove and Wayne N. Bushell. The chi square periodogram: Its utility for analysis of circadian rhythms. *Journal of Theoretical Biology*, 72(1):131–160, May 1978.

[8] Jordi Garcia-Ojalvo, Michael B Elowitz, and Steven H Strogatz. Modeling a synthetic multicellular clock: repressilators coupled by quorum sensing. *Proceedings of the National Academy of Sciences of the United States of America*, 101(30):10955–10960, 2004.

[9] Mathew D Edwards, Marco Brancaccio, Johanna E Chesham, Elizabeth S Maywood, and Michael H Hastings. Rhythmic expression of cryptochrome induces the circadian clock of arrhythmic suprachiasmatic nuclei through arginine vasopressin signaling. *Proceedings of the National Academy of Sciences*, 113(10):2732–2737, 2016.

[10] Daisuke Ono, Sato Honma, and Ken-Ichi Honma. Cryptochromes are critical for the development of coherent circadian rhythms in the mouse suprachiasmatic nucleus. *Nat Commun*, 4:1666, 2013.

[11] Pål O. Westermark, David K. Welsh, Hitoshi Okamura, and Hanspeter Herzel. Quantification of circadian rhythms in single cells. *PLoS Comput Biol*, 5(11):e1000580, November 2009.

[12] Arthur T Winfree. *The geometry of biological time*, volume 12. Springer Science & Business Media, 2001.

[13] Alexis B. Webb, Nikhil Angelo, James E. Huettner, and Erik D. Herzog. Intrinsic, nondeterministic circadian rhythm generation in identified mammalian neurons. *Proceedings of the National Academy of Sciences*, 106(38):16493–16498, Sep 2009.

[14] Isao T Tokuda, Daisuke Ono, Bharath Ananthasubramaniam, Sato Honma, Ken-Ichi Honma, and Hanspeter Herzel. Coupling controls the synchrony of clock cells in development and knockouts. *Biophysical journal*, 109(10):2159–2170, 2015.

[15] Daisuke Ono, Sato Honma, and Ken-Ichi Honma. Differential roles of avp and vip signaling in the postnatal changes of neural networks for coherent circadian rhythms in the scn. *Science advances*, 2(9):e1600960, 2016.

[16] Yuriko Ban, Yasufumi Shigeyoshi, and Hitoshi Okamura. Development of vasoactive intestinal peptide mRNA rhythm in the rat suprachiasmatic nucleus. *J. Neurosci.*, 17(10):3920–3931, May 1997.

[17] Kazuyuki Shinohara, Toshiya Funabashi, and Fukuko Kimura. Temporal profiles of vasoactive intestinal polypeptide precursor mrna and its receptor mrna in the rat suprachiasmatic nucleus. *Molecular brain research*, 63(2):262–267, 1999.
