# Supplementary figures and images for: Coherency of circadian rhythms in the SCN is governed by the interplay of two coupling factors
Source: PLoS Comput Biol. 2018 Dec 10;14(12):e1006607. doi: 10.1371/journal.pcbi.1006607 (PMC6301697; doi:10.1371/journal.pcbi.1006607)

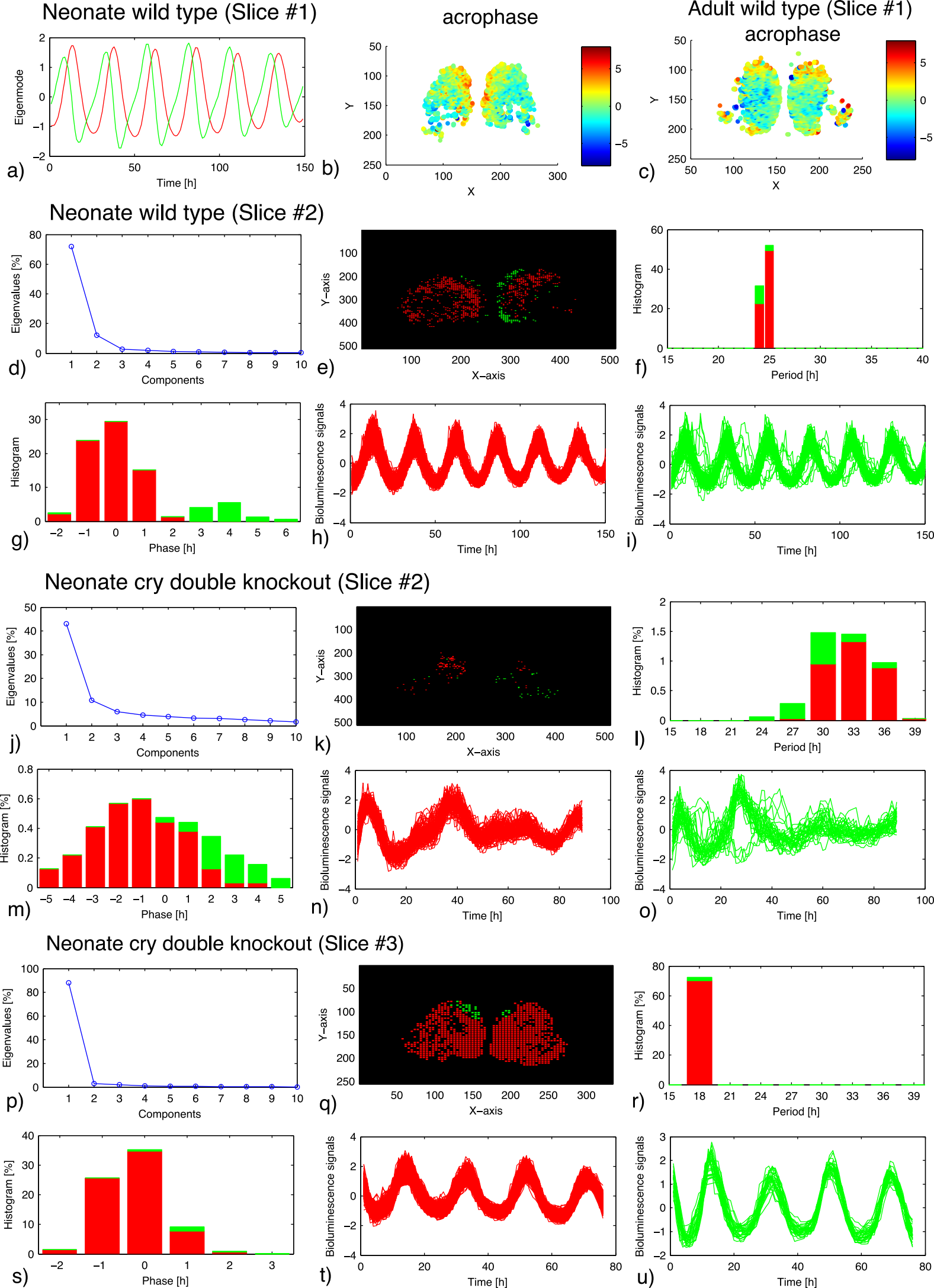

Supplement: S1 Fig — (a): First (red) and second (green) eigenmodes of the empirical orthogonal function. (b),(c): Acrophases of whole cells on neonate wild–type slice #1 (b) and adult wild–type slice #1 (c). (d),(j),(p): Eigenvalues of the empirical orthogonal function. (e),(k),(q): Location of the cells classified as first (red) and second (green) empirical modes. (f),(l),(r): Period distribution of the cells classified as first (red) and second (green) empirical modes. (g),(m),(s): Acrophase distribution of the cells classified as first (red) and second (green) empirical modes. (h),(i),(n),(o),(t),(u): Bioluminescence traces of the cells classified as first (red) and second (green) empirical modes. (PDF) [file pcbi.1006607.s002.pdf]

# Neonate triple knockout (Slice #2)

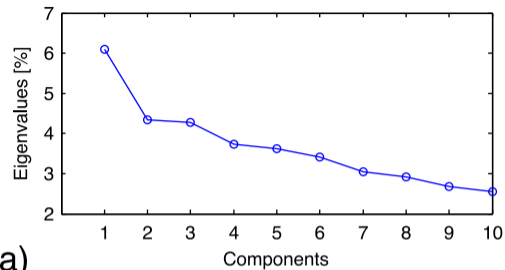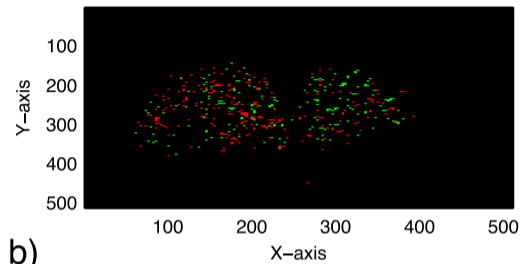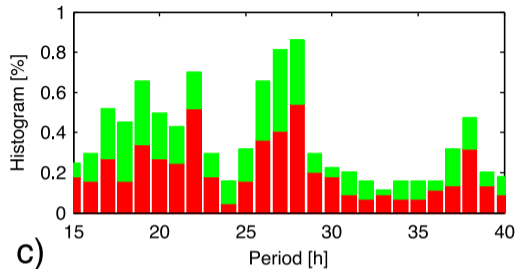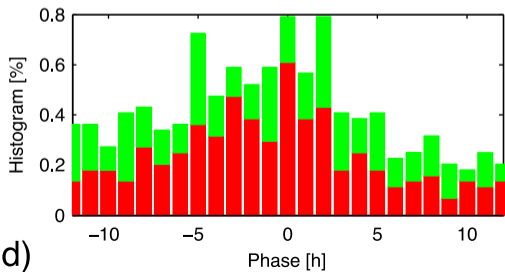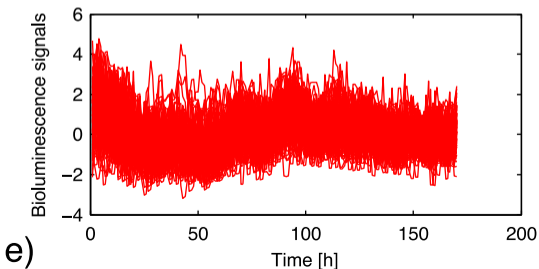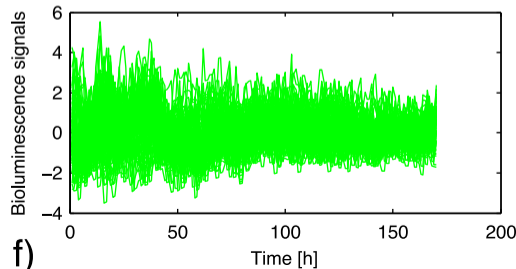

Supplement: S2 Fig — (a): Eigenvalues of the empirical orthogonal function. (b): Location of the cells classified as first (red) and second (green) empirical modes. (c): Period distribution of the cells classified as first (red) and second (green) empirical modes. (d): Acrophase distribution of the cells classified as first (red) and second (green) empirical modes. (e),(f): Bioluminescence traces of the cells classified as first (red) and second (green) empirical modes. (PDF) [file pcbi.1006607.s003.pdf]

### Adult wild type (Slice #1)

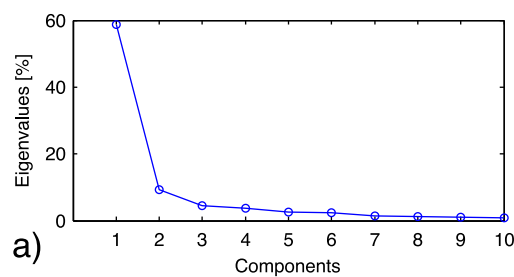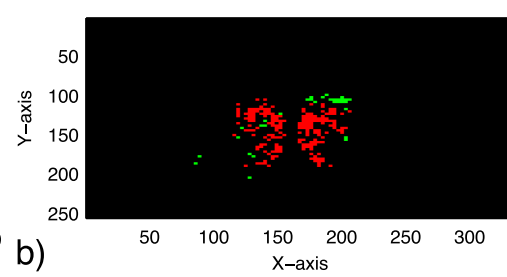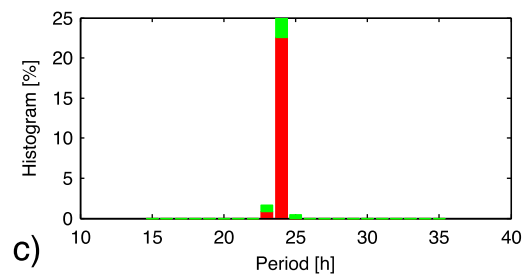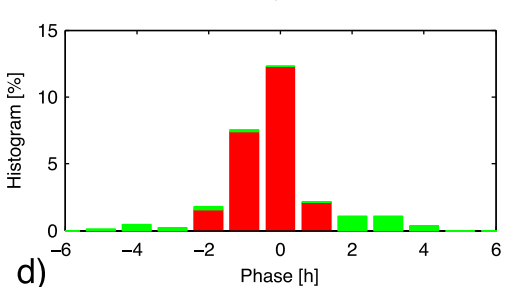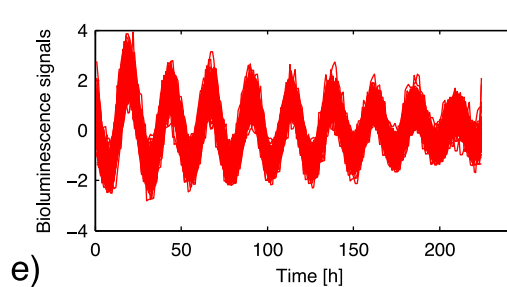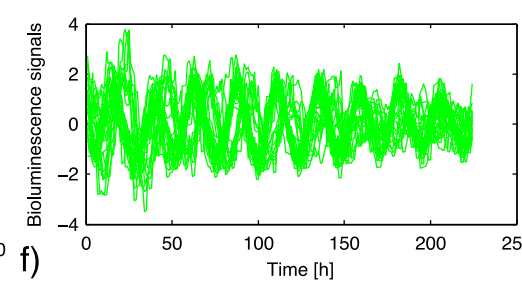

### Adult cry double knockout (Slice #1)

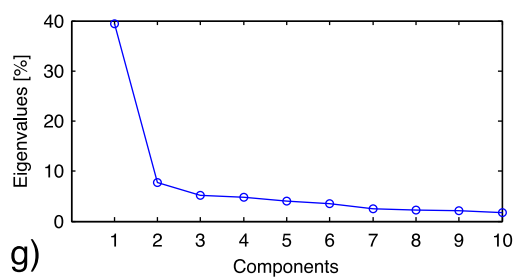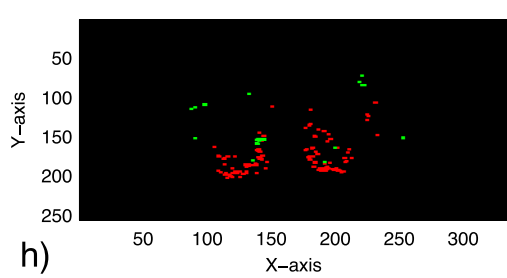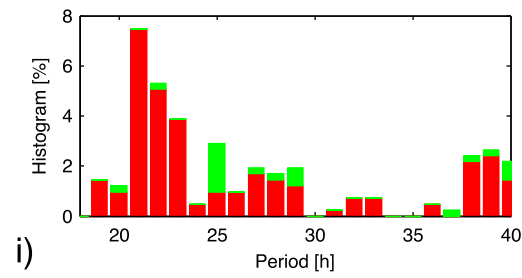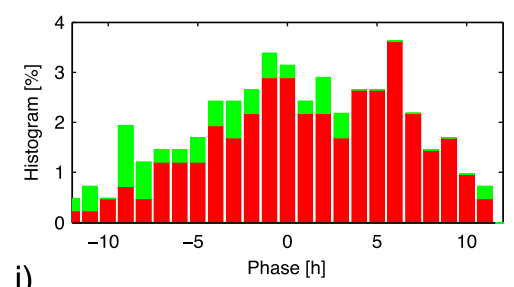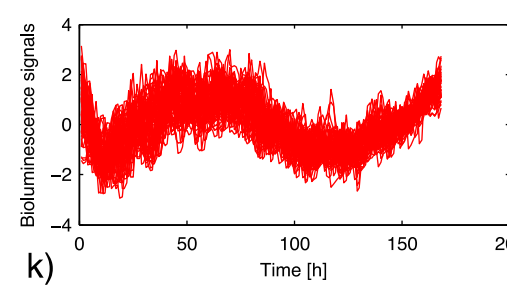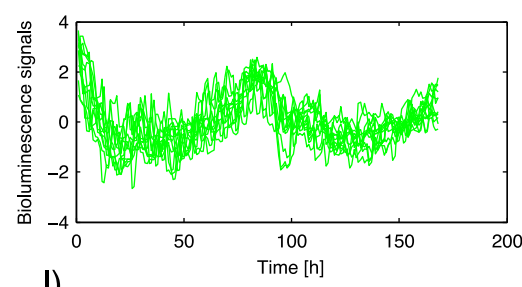

### Adult triple knockout (Slice #1)

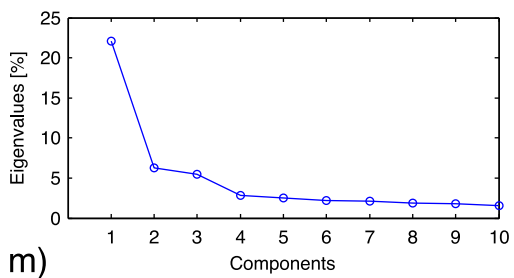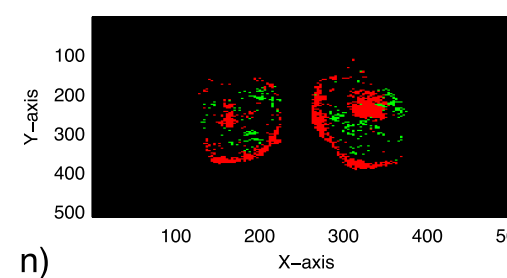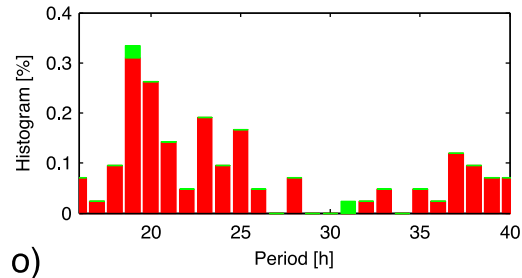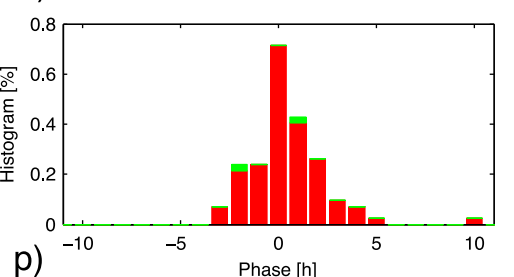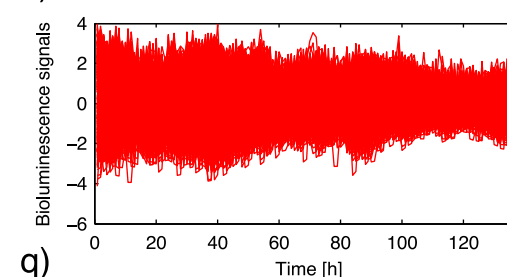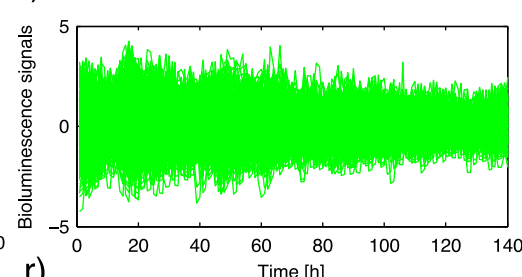

Supplement: S3 Fig — (a),(g),(m): Eigenvalues of the empirical orthogonal function. (b),(h),(n): Location of the cells classified as first (red) and second (green) empirical modes. (c),(i),(o): Period distribution of the cells classified as first (red) and second (green) empirical modes. (d),(j),(p): Acrophase distribution of the cells classified as first (red) and second (green) empirical modes. (e),(f),(k),(l),(q),(r): Bioluminescence traces of the cells classified as first (red) and second (green) empirical modes. (PDF) [file pcbi.1006607.s004.pdf]

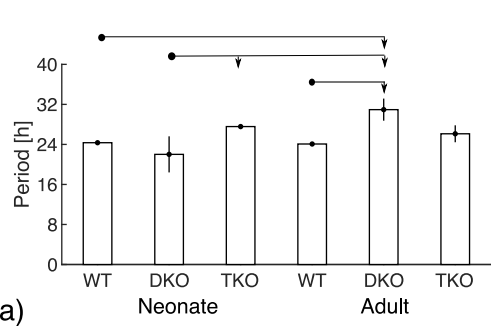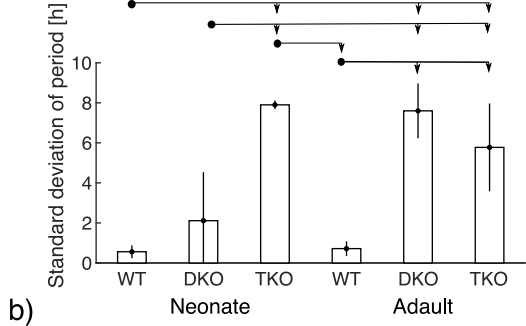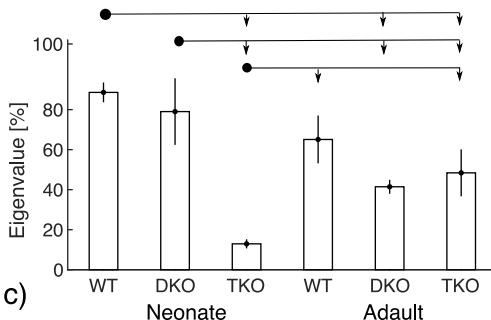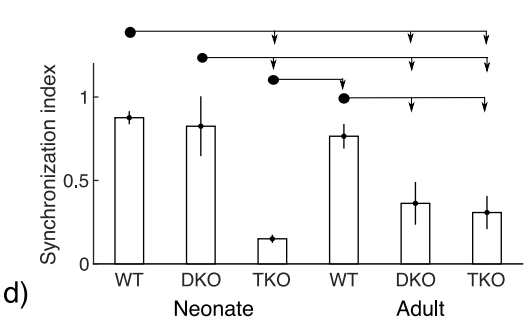

Supplement: S4 Fig — One-way ANOVA revealed significant main effect (p < 0.01) for all four quantities. Post hoc comparisons using Fisher’s least significant difference (p < 0.01) indicate pairs of group means that differ from each other (each pair indicated with a combination of filled circle and arrow). (PDF) [file pcbi.1006607.s005.pdf]

## Wild type dispersed cell data

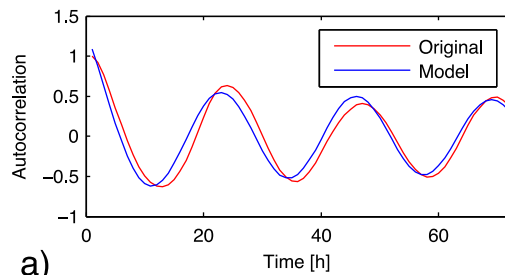

a)

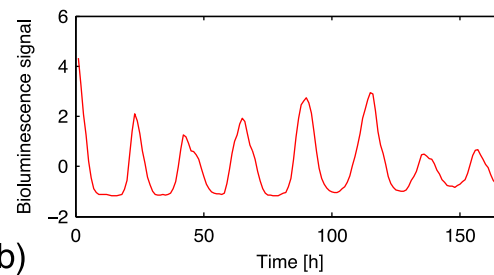

b)

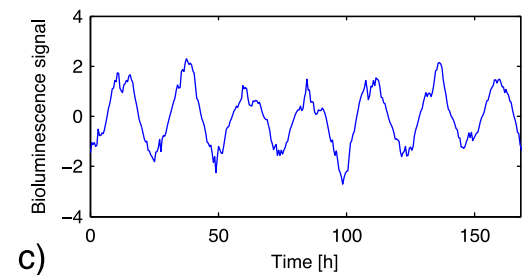

c)

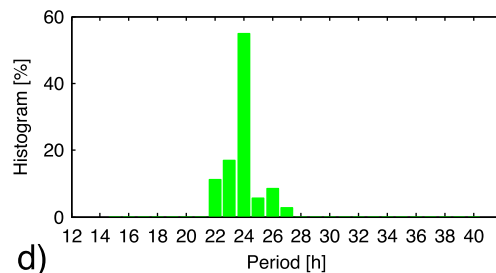

d)

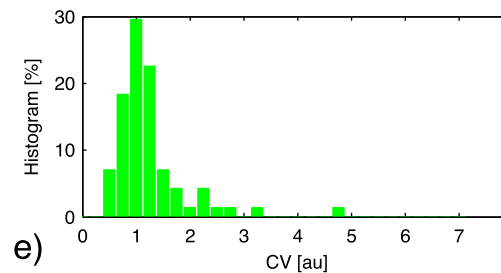

e)

## Cry double knockout dispersed cell data

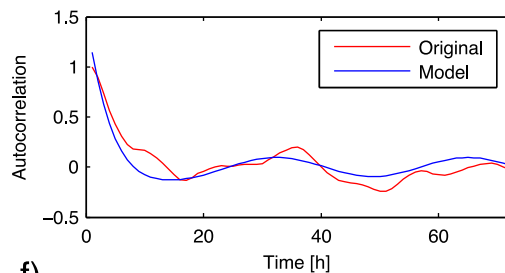

f)

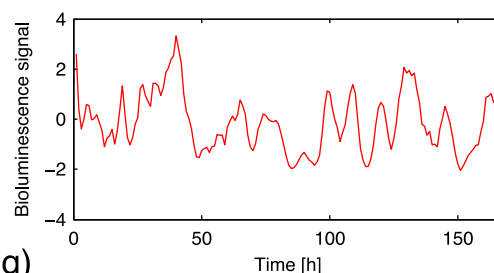

g)

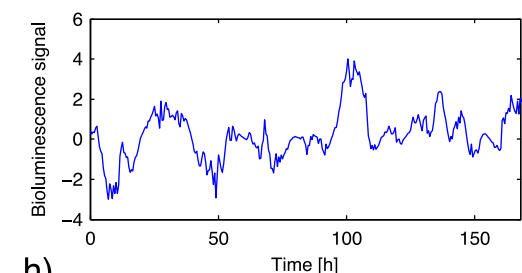

h)

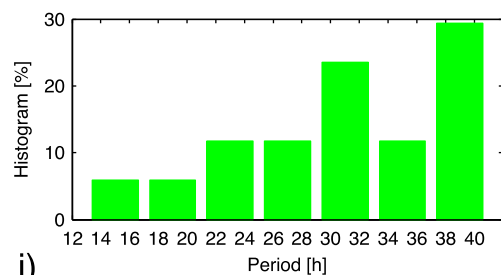

i)

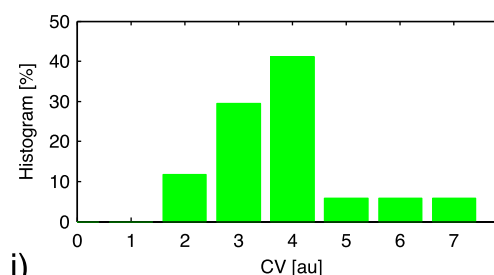

j)

Supplement: S9 Fig — (a), (f): Autocorrelation functions of an experimental data (red) and the corresponding amplitude–phase model (blue). (b), (g): Detrended and normalized bioluminescence signals. (c), (h): Simulated signal by the stochastic amplitude model with estimated parameters. (d), (i): Distribution of period estimated from dispersed SCN cell cultures. (e), (j): Distribution of coefficient of variation, CV, estimated from dispersed SCN cell cultures. (PDF) [file pcbi.1006607.s010.pdf]
