# Supplementary material for: Coherency of circadian rhythms in the SCN is governed by the interplay of two coupling factors
Source: PLoS Comput Biol. 2018 Dec 10;14(12):e1006607. doi: 10.1371/journal.pcbi.1006607 (PMC6301697; doi:10.1371/journal.pcbi.1006607)
Supplement: S5 Fig — A cocktail of AVP receptor antagonists (SR49059: AVP receptor V1a antagonist, SSR149415: AVP receptor V1a and V1b antagonists) was applied to the cultured SCN slices in (d–f),(j–l). (a),(d),(g),(j): Eigenvalues of the empirical orthogonal function. (b),(e),(h),(k): Location of the cells classified as first (red) and second (green) empirical modes. (c),(f),(i),(l): Period distribution of the cells classified as first (red) and second (green) empirical modes. (PDF) [file pcbi.1006607.s006.pdf]

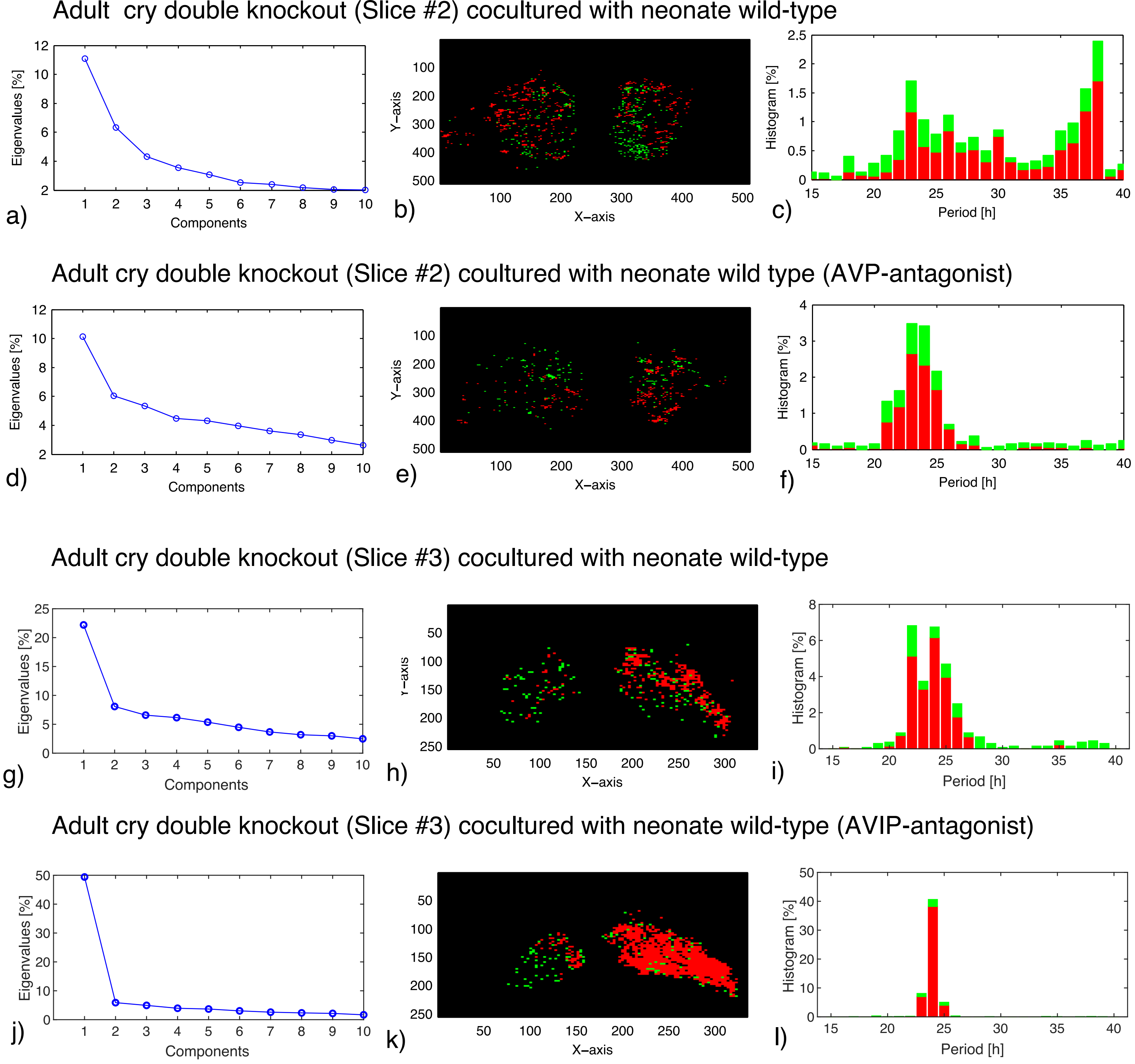
Adult cry double knockout (Slice #2) cocultured with neonate wild-type
Eigenvalues [%]
Components
a)
Y–axis
X–axis
b)
Histogram [%]
Period [h]
c)
Adult cry double knockout (Slice #2) coultured with neonate wild type (AVP-antagonist)
Eigenvalues [%]
Components
d)
Y–axis
X–axis
e)
Histogram [%]
Period [h]
f)
Adult cry double knockout (Slice #3) cocultured with neonate wild-type
Eigenvalues [%]
Components
g)
Y–axis
X–axis
h)
Histogram [%]
Period [h]
i)
Adult cry double knockout (Slice #3) cocultured with neonate wild-type (AVIP-antagonist)
Eigenvalues [%]
Components
j)
X–axis
k)
Histogram [%]
Period [h]
l)
