# Supplementary material for: Coherency of circadian rhythms in the SCN is governed by the interplay of two coupling factors
Source: PLoS Comput Biol. 2018 Dec 10;14(12):e1006607. doi: 10.1371/journal.pcbi.1006607 (PMC6301697; doi:10.1371/journal.pcbi.1006607)
Supplement: S6 Fig — AVP receptor antagonists were applied in (g–l). (PDF) [file pcbi.1006607.s007.pdf]

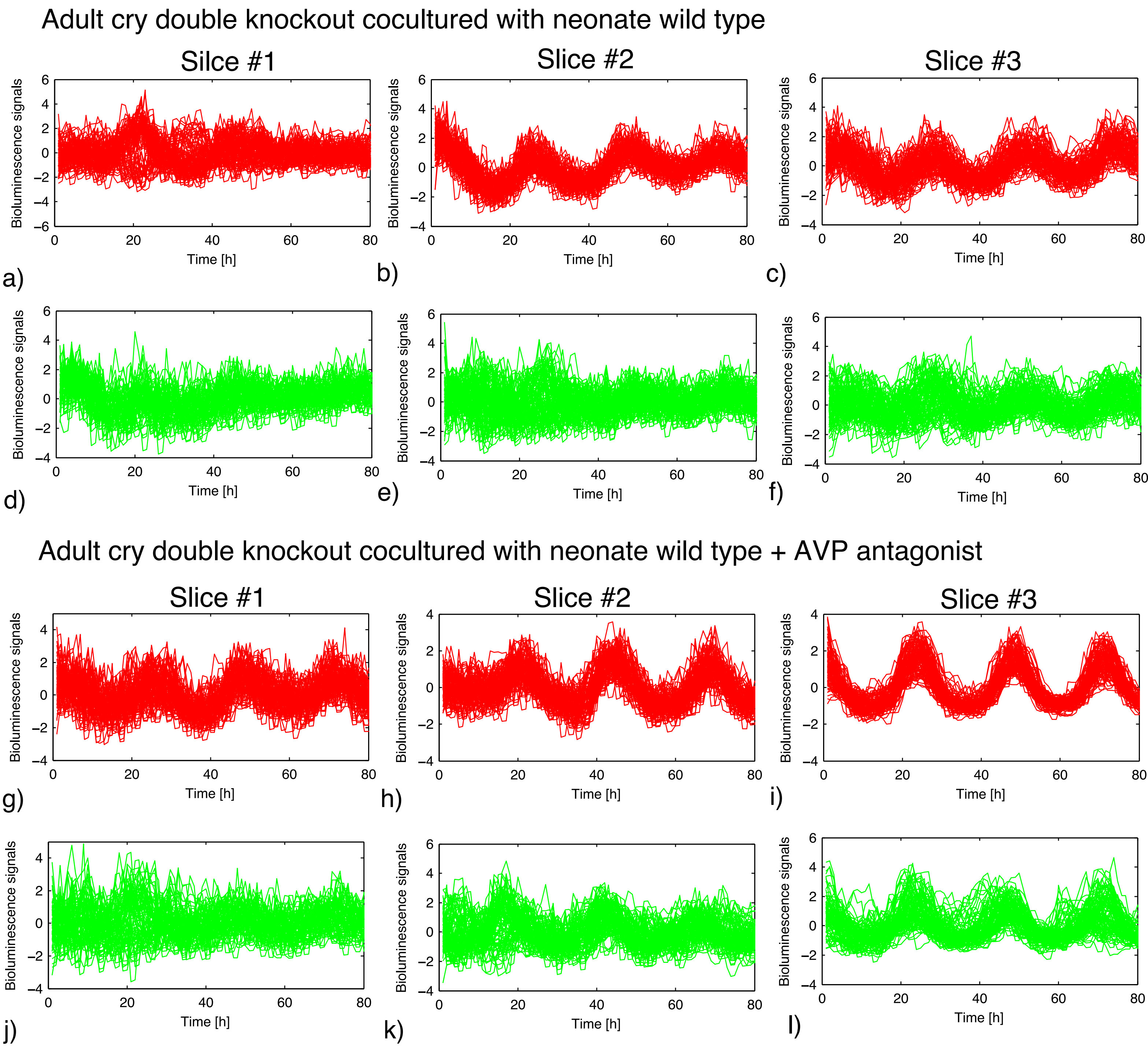
Adult cry double knockout cocultured with neonate wild type
Silce #1
Slice #2
Slice #3
Bioluminescence signals
Time [h]
a)
b)
c)
d)
e)
f)
Adult cry double knockout cocultured with neonate wild type + AVP antagonist
Slice #1
Slice #2
Slice #3
g)
h)
i)
j)
k)
l)
