# Supplementary material for: Coherency of circadian rhythms in the SCN is governed by the interplay of two coupling factors
Source: PLoS Comput Biol. 2018 Dec 10;14(12):e1006607. doi: 10.1371/journal.pcbi.1006607 (PMC6301697; doi:10.1371/journal.pcbi.1006607)

Adult triple knockout (Slice #2) cocultured with neonate wild-type

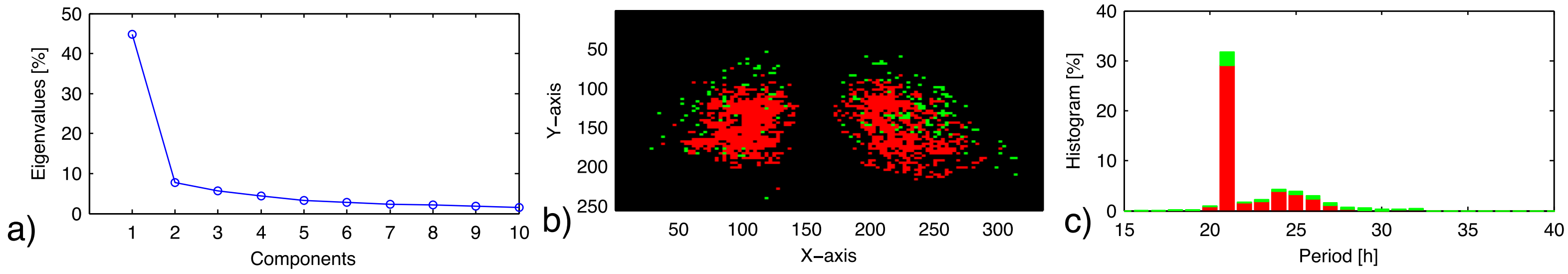

Adult triple knockout (Slice #2) coultured with neonate wild type (AVP-antagonist)

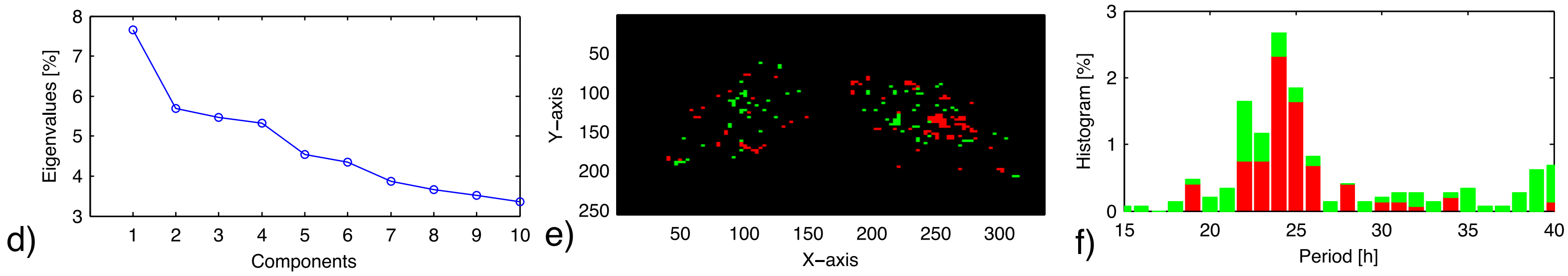

Adult triple knockout (Slice #3) cocultured with neonate wild-type

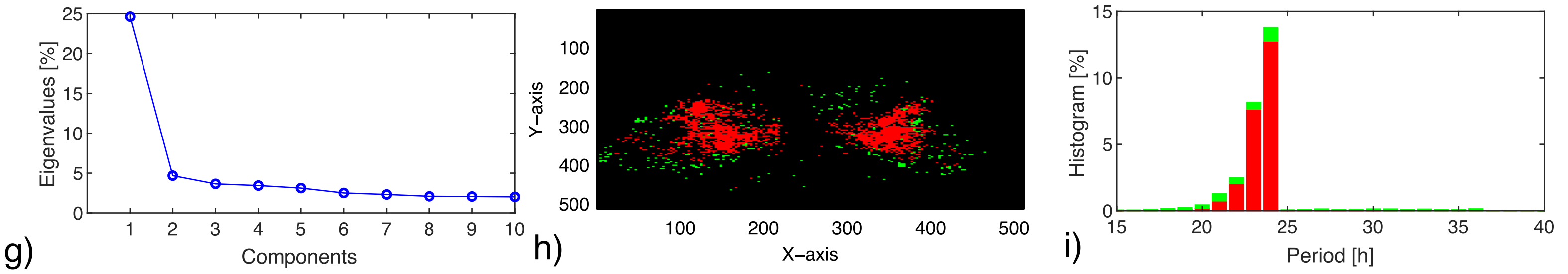

Adult triple knockout (Slice #3) cocultured with neonate wild-type (AVP-antagonist)

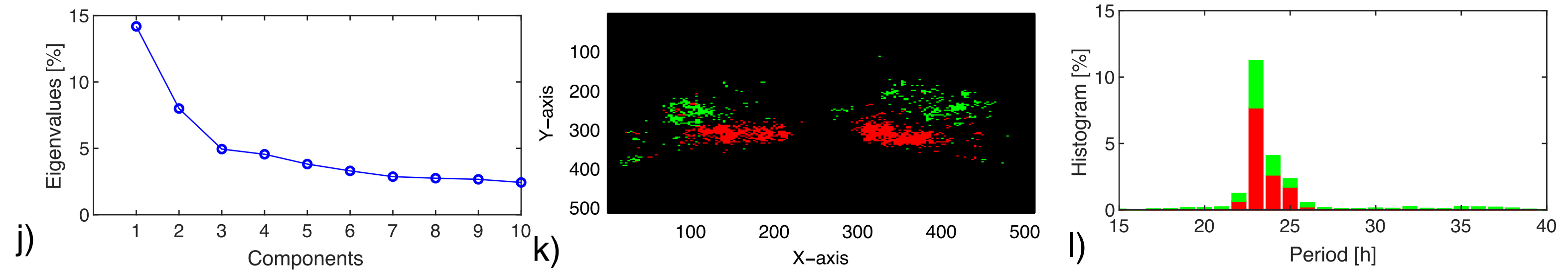

Supplement: S7 Fig — A cocktail of AVP receptor antagonists (SR49059: AVP receptor V1a antagonist, SSR149415: AVP receptor V1a and V1b antagonists) was applied to the cultured SCN slices in (d–f),(j–l). (a),(d),(g),(j): Eigenvalues of the empirical orthogonal function. (b),(e),(h),(k): Location of the cells classified as first (red) and second (green) empirical modes. (c),(f),(i),(l): Period distribution of the cells classified as first (red) and second (green) empirical modes. (PDF) [file pcbi.1006607.s008.pdf]
