# Supplementary material for: Coherency of circadian rhythms in the SCN is governed by the interplay of two coupling factors
Source: PLoS Comput Biol. 2018 Dec 10;14(12):e1006607. doi: 10.1371/journal.pcbi.1006607 (PMC6301697; doi:10.1371/journal.pcbi.1006607)
Supplement: S10 Fig — (a): Dependence of the synchronization index R on the attenuation factors avip∈[0, 1], aavp∈[0, 1] was computed for the network of wild-type cells. (b): Dependence of the synchronization index R on the attenuation factors avip∈[0, 0.7], aavp∈[0, 1] was computed for the network of double knockout cells. (c): For the network of double knockout cells, synchronization indices, Rl and Rr, are computed separately for left and right sides of the SCN and their average is drawn. (d): Entrainment property of the network of double knockout cells (avip = 0.1, aavp = 0.1) forced by VIP and AVP signals Ivip = 0.01 and Iavp∈[0, 0.01]. Dependence of the synchronization index R on the phase–delay ϕ and the strength of AVP signaling Iavp is plotted. (PDF) [file pcbi.1006607.s011.pdf]

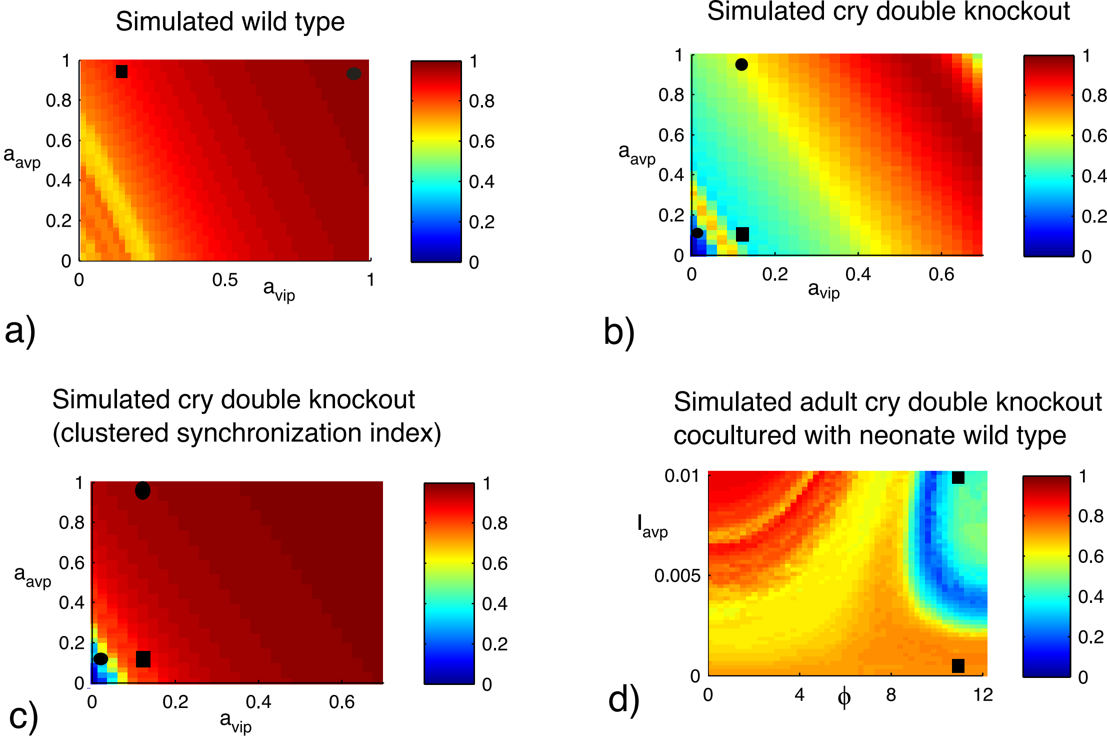
Simulated wild type
a_avp
a_vip
a)
Simulated cry double knockout
a_avp
a_vip
b)
Simulated cry double knockout
(clustered synchronization index)
a_avp
a_vip
c)
Simulated adult cry double knockout
cocultured with neonate wild type
I_avp
ϕ
d)
