# Supplementary material for: Coherency of circadian rhythms in the SCN is governed by the interplay of two coupling factors
Source: PLoS Comput Biol. 2018 Dec 10;14(12):e1006607. doi: 10.1371/journal.pcbi.1006607 (PMC6301697; doi:10.1371/journal.pcbi.1006607)
Supplement: S11 Fig — (a),(g),(m): Eigenvalues of the EOF. (b),(h),(n): Location of the cells classified as first (red) and second (green) components. (c),(i),(o): Period distribution of the cells classified as the two principal components. (d),(j),(p): Acrophase distribution of the cells classified as the two principal components. (e),(f),(k),(l),(q),(r): Simulated traces of the cells classified as the principal components. (PDF) [file pcbi.1006607.s012.pdf]

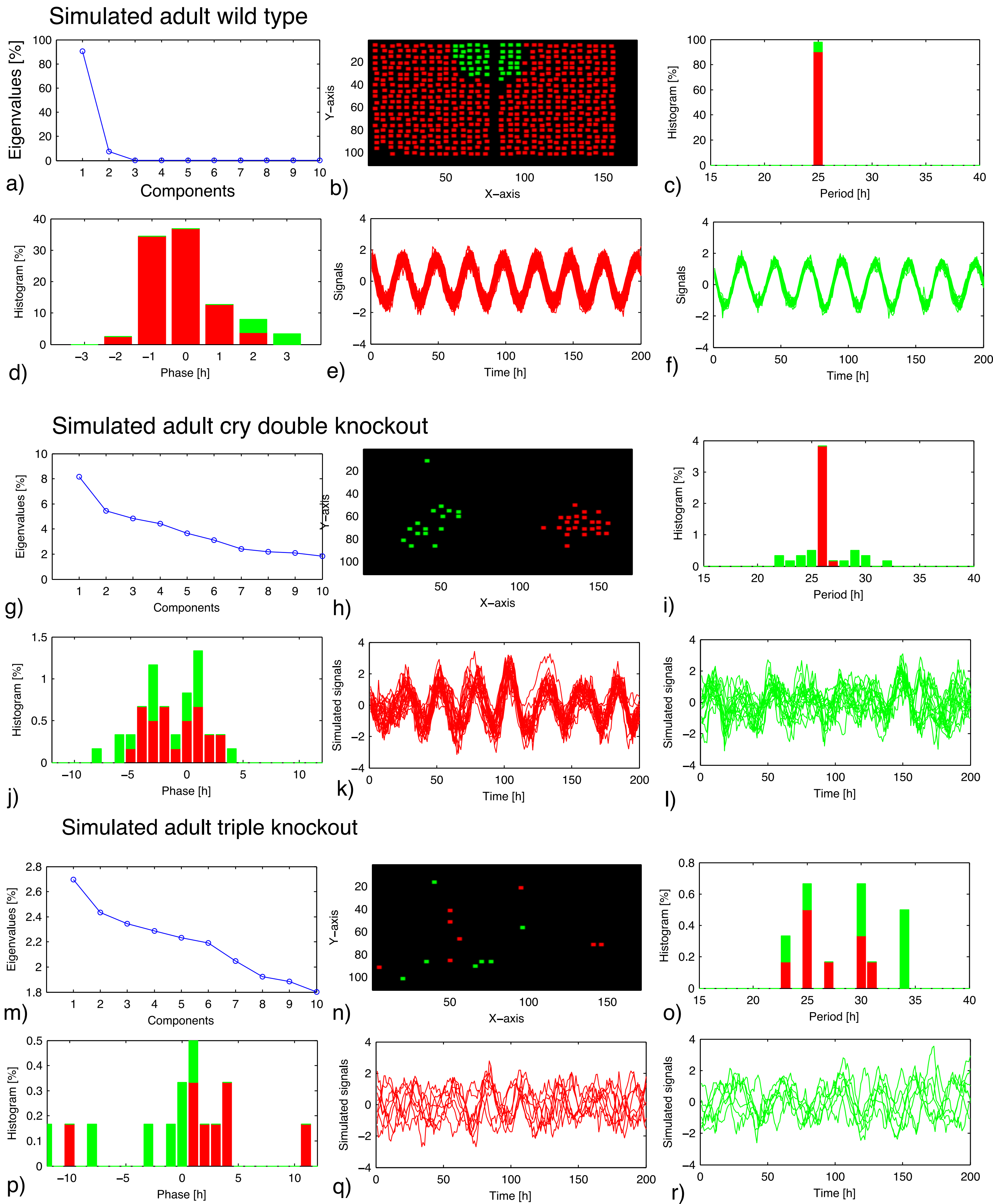

Simulated adult wild type
Eigenvalues [%]
Components
a)
Y–axis
X–axis
b)
Histogram [%]
Period [h]
c)
Histogram [%]
Phase [h]
d)
Signals
Time [h]
e)
Signals
Time [h]
f)
Simulated adult cry double knockout
Eigenvalues [%]
Components
g)
Y–axis
X–axis
h)
Histogram [%]
Period [h]
i)
Histogram [%]
Phase [h]
j)
Simulated signals
Time [h]
k)
Simulated signals
Time [h]
l)
Simulated adult triple knockout
Eigenvalues [%]
Components
m)
Y–axis
X–axis
n)
Histogram [%]
Period [h]
o)
Histogram [%]
Phase [h]
p)
Simulated signals
Time [h]
q)
Simulated signals
Time [h]
r)
