# Supplementary material for: Coherency of circadian rhythms in the SCN is governed by the interplay of two coupling factors
Source: PLoS Comput Biol. 2018 Dec 10;14(12):e1006607. doi: 10.1371/journal.pcbi.1006607 (PMC6301697; doi:10.1371/journal.pcbi.1006607)
Supplement: S12 Fig — Pharmacological treatment with AVP antagonists is assumed as Iavp = 0 in b),(d),(f),(h). (PDF) [file pcbi.1006607.s013.pdf]

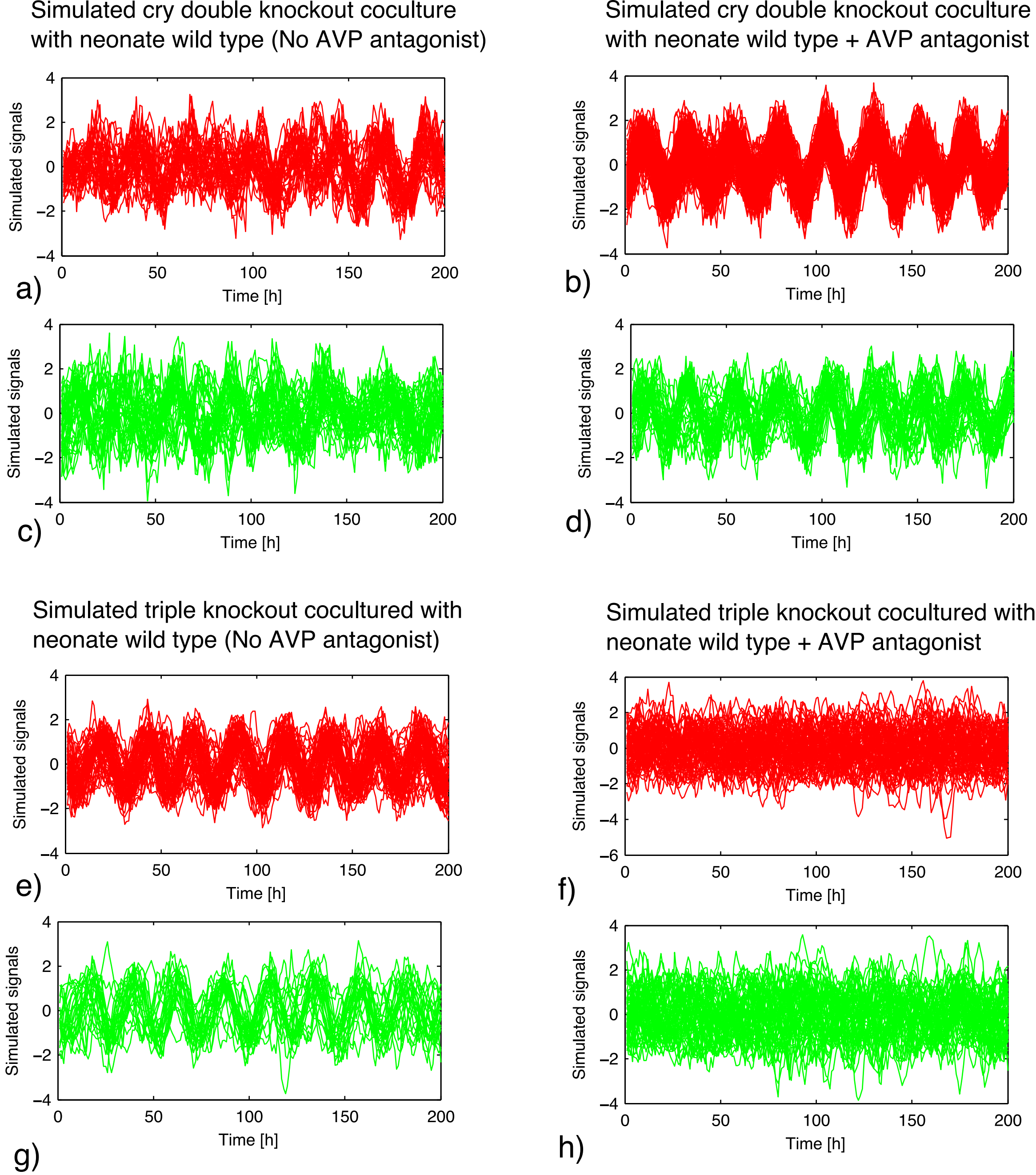
Simulated cry double knockout coculture
with neonate wild type (No AVP antagonist)
Simulated cry double knockout coculture
with neonate wild type + AVP antagonist
Simulated signals
Time [h]
a)
b)
c)
d)
Simulated triple knockout cocultured with
neonate wild type (No AVP antagonist)
Simulated triple knockout cocultured with
neonate wild type + AVP antagonist
e)
f)
g)
h)
0
50
100
150
200
4
2
0
−2
−4
−6
