# Supplementary material for: Coherency of circadian rhythms in the SCN is governed by the interplay of two coupling factors
Source: PLoS Comput Biol. 2018 Dec 10;14(12):e1006607. doi: 10.1371/journal.pcbi.1006607 (PMC6301697; doi:10.1371/journal.pcbi.1006607)
Supplement: S1 Table — Average and standard deviation of the period estimated by the chi–square periodogram (significance level of 1%) [71] are indicated. Summation of the normalized first and second eigenvalues was calculated by the EOF analysis. Synchronization index R was also computed, where the average and standard deviation are for 24 time points. (PDF) [file pcbi.1006607.s014.pdf]

Phenotype	Slice	Period	$\Omega_1 + \Omega_2$	Synchrony
Cry1,2$^{+/+}$/*Vipr2*$^{+/+}$	# 1	24.4±0.5 h	90.5 %	0.89±0.01
	# 2	24.6±0.6 h	84.0 %	0.86±0.02
	# 3	24.7±0.5 h	82.6 %	0.82±0.02
	# 4	24.0±0.1 h	96.4 %	0.94±0.01
	# 5	24.0±1.1 h	89.5 %	0.87±0.03
Cry1,2$^{-/-}$/*Vipr2*$^{+/+}$	# 1	28.0±6.8 h	50.5 %	0.47±0.11
	# 2	26.8±5.5 h	53.9 %	0.58±0.07
	# 3	19.0±0.2 h	90.1 %	0.97±0.003
	# 4	21.5±0.5 h	87.9 %	0.94±0.02
	# 5	17.8±2.2 h	84.5 %	0.89±0.02
	# 6	23.4±0.8 h	75.4 %	0.88±0.09
	# 7	21.6±0.5 h	98.3 %	0.99±0.001
	# 8	18.0±0.4 h	91.5 %	0.88±0.005
Cry1,2$^{-/-}$/*Vipr2*$^{-/-}$	# 1	28.1±7.6 h	12.5 %	0.15±0.04
	# 2	27.7±8.1 h	10.4 %	0.12±0.03
	# 3	26.9±8.0 h	15.9 %	0.18±0.06
