# Supplementary material for: Coherency of circadian rhythms in the SCN is governed by the interplay of two coupling factors
Source: PLoS Comput Biol. 2018 Dec 10;14(12):e1006607. doi: 10.1371/journal.pcbi.1006607 (PMC6301697; doi:10.1371/journal.pcbi.1006607)
Supplement: S2 Table — Average and standard deviation of the period estimated by the chi–square periodogram (significance level of 1%) [71] are indicated. Summation of the normalized first and second eigenvalues was calculated by the EOF analysis. Synchronization index R was also computed, where the average and standard deviation are for 24 time points. (PDF) [file pcbi.1006607.s015.pdf]

Phenotype	Slice	Period	$\Omega_1 + \Omega_2$	Synchrony
Cry1,2$^{+/+}$/*Vipr2*$^{+/+}$	# 1	23.9±0.6 h	69.0 %	0.81±0.03
	# 2	24.0±0.3 h	80.0 %	0.85±0.06
	# 3	24.1±0.3 h	75.8 %	0.85±0.02
	# 4	24.2±1.3 h	63.0 %	0.71±0.09
	# 5	24.5±1.0 h	59.2 %	0.67±0.04
	# 6	23.8±0.8 h	43.5 %	0.70±0.03
Cry1,2$^{-/-}$/*Vipr2*$^{+/+}$	# 1	30.0±8.2 h	47.1 %	0.25±0.08
	# 2	28.5±8.0 h	39.7 %	0.22±0.13
	# 3	30.8±5.3 h	41.3 %	0.49±0.11
	# 4	34.5±8.9 h	37.7 %	0.49±0.11
Cry1,2$^{-/-}$/*Vipr2*$^{-/-}$	# 1	24.9±7.4 h	28.3 %	0.29±0.07
	# 2	24.2±2.1 h	57.3 %	0.27±0.11
	# 3	28.5±7.5 h	52.5 %	0.20±0.07
	# 4	26.9±6.1 h	55.5 %	0.47±0.21
