# Supplementary material for: Coherency of circadian rhythms in the SCN is governed by the interplay of two coupling factors
Source: PLoS Comput Biol. 2018 Dec 10;14(12):e1006607. doi: 10.1371/journal.pcbi.1006607 (PMC6301697; doi:10.1371/journal.pcbi.1006607)
Supplement: S3 Table — Condition, under which a cocktail of AVP receptor antagonists was applied to the slice, was compared with the control condition. Period, estimated by the chi–square periodogram, summation of the normalized first and second eigenvalues, calculated by the EOF analysis, and synchronization index are summarized. (PDF) [file pcbi.1006607.s016.pdf]

Recipient	Slice	Antagonist	Period	$\Omega_1 + \Omega_2$	Synchrony
Cry1,2$^{-/-}$ *Vipr2*$^{+/+}$	# 1	No antagonist	26.9±7.1 h	19.0 %	0.27±0.04
		+V1a,b antagonist	25.0±4.9 h	30.9 %	0.48±0.09
	# 2	No antagonist	27.9±7.0 h	17.4 %	0.12±0.06
		+V1a,b antagonist	25.8±6.0 h	16.1 %	0.25±0.08
	# 3	No antagonist	26.4±6.4 h	30.7 %	0.47±0.09
		+V1a,b antagonist	24.3±3.1 h	55.7 %	0.75±0.03
Cry1,2$^{-/-}$ *Vipr2*$^{-/-}$	# 1	No antagonist	24.5±5.4 h	26.2 %	0.35±0.03
		+V1a,b antagonist	26.8±6.9 h	8.4 %	0.07±0.02
	# 2	No antagonist	22.8±3.4 h	52.4 %	0.63±0.05
		+V1a,b antagonist	26.3±6.5 h	13.3 %	0.14±0.03
	# 3	No antagonist	23.7±4.2 h	27.4 %	0.40±0.13
		+V1a,b antagonist	25.9±6.1 h	22.2 %	0.23±0.06
